# Hippocampus-targeted BDNF gene therapy to rescue cognitive impairments of Alzheimer's disease in multiple mouse models

**DOI:** 10.1016/j.gendis.2025.101649

**Published:** 2025-04-22

**Authors:** Siqi Tang, Wenshu Luo, Shihao Wu, Meng Yuan, Jiashuo Wen, Guoshen Zhong, Leshan Shen, Wei Jiang, Cheng Cheng, Xia Wu, Xiao Xiao

**Affiliations:** a School of Pharmacy, East China University of Science and Technology, Shanghai 200237, China; b Belief Biomed Inc, Shanghai 200237, China; c School of Medicine, Yunnan University, Kunming, Yunnan 650106, China; d School of Bioengineering, East China University of Science and Technology, Shanghai 200237, China

**Keywords:** AAVT42, Alzheime's disease, Brain-derived neurotrophic factor, Cognitive impairment, Hippocampal RNA sequencing

## Abstract

Brain-derived neurotrophic factor (BDNF) can protect neurons from apoptosis and maintain normal synaptic structures, indicating a significant potential for Alzheimer's disease (AD) treatment. However, the method of *in vivo* BDNF delivery requires further optimization, and the therapeutic efficacy of BDNF in AD animal models needs to be further evaluated. Here, we demonstrated that a newly engineered adeno-associated virus (AAV) serotype termed AAVT42 showed better tropism for neurons than AAV9 in the central nervous system (CNS). We analyzed the therapeutic potentials of AAVT42-delivered BDNF in three AD mouse models: amyloid precursor protein/presenilin-1 (APP/PS1), rTg4510, and 3 × Tg. Long-term BDNF expression in the hippocampus mitigated neuronal degeneration or loss in these AD mice, and alleviated their cognitive impairment, with no discernible effect on amyloid-β deposition or tau phosphorylation. Furthermore, transcriptomic analysis in 3 × Tg mice revealed that BDNF orchestrated the up-regulation of genes associated with neuronal structural organization and synaptic transmissions, such as Neuropeptide Y (*Npy*), Corticotropin-releasing hormone (*Crh*), Tachykinin precursor 1 (*Tac1*), and the down-regulation of Bone morphogenetic proteins (*Bmps*). Our study highlighted the efficacy of AAVT42 in gene delivery to CNS and validated the therapeutic benefits of BDNF in treating AD, which will be useful for future translational research on AD treatment using an AAV delivery system.

## Introduction

Degeneration of memory and cognitive function is the major symptom of Alzheimer's disease (AD), resulting from the progressive loss of neuron function. The U.S. FDA-approved major treatments for AD include anticholinesterase inhibitors and anti-glutamatergic drugs. Although these therapies temporarily prevent or delay neurotoxicity and disease progression, they cannot halt or reverse the symptoms.^1^^,^^2^ In addition, therapeutics targeting the two hallmark features of AD, amyloid-beta (Aβ) plaques,^3^ and neurofibrillary tangle,^4^ have been developed. Recent clinical trials have shown that monoclonal antibodies can reduce Aβ accumulation in the brain and slow disease progression in the early-stage AD patients.^5^, ^6^, ^7^ While this strategy emphasizes the critical role of Aβ in AD pathogenesis, the clinical benefits of antibody-modifying therapies have been limited, and the disease continues to progress.^8^ With our developing understanding of AD pathophysiology, more and more investigations focused on early diagnosis and preventive treatment.

Brain-derived neurotrophic factor (BDNF) plays essential roles in the central nervous system (CNS) development, neuronal structure maintenance, neuron–glial interactions, neuron homeostasis, and activity-dependent structural remodeling in adulthood,^9^^,^^10^ and it is especially beneficial for neuron survival after brain injury due to its anti-apoptotic effects.^11^^,^^12^ Significantly lower BDNF expression has been reported in the cortex, hippocampus, cerebrospinal fluid, and serum^13^, ^14^, ^15^, ^16^ of AD patients and model rodents.^17^^,^^18^ Previous studies have demonstrated that transgenic BDNF expression in the brain can promote neurogenesis, elicit anxiolytic-like activities,^19^ and alleviate cognitive impairment in AD rodent models.^20^^,^^21^ Therefore, finding an efficient method to deliver transgenes, such as BDNF, into the brain is crucial for improving therapeutic outcomes.

Over the past decades, great breakthroughs and progress have been made in the clinical application of recombinant adeno-associated virus (rAAV) in treating CNS, ocular, muscular, and hepatic diseases.^22^ With advancements in evolution, screening methods, and the development of various delivery routes, including intraparenchymal, intrathecal, and intracerebroventricular injections,^23^^,^^24^ novel rAAV capsids with enhanced delivery efficiency, tissue specificity, cell tropism, and improved safety profiles^25^^,^^26^ have been increasingly applied in neurological disorder treatment. In recent years, the U.S. FDA has approved several AAV-based therapies, including AAV2 gene therapy for Leber congenital amaurosis and AAV9 for spinal muscular atrophy (SMA).^27^^,^^28^ AAV-based gene therapy for treating more complex neurodegenerative diseases, such as AD, has also garnered increasing attention. Early attempts to deliver nerve growth factor to the cortex demonstrated a favorable safety profile for AAV-mediated delivery in AD patients.^29^^,^^30^ Delivery of BDNF encoding genes into the rat hippocampus or the mice entorhinal cortex via AAV or lentivirus has been shown to protect neurons and restore learning and memory deficits,^31^^,^^32^ thereby driving further research on its potential therapeutic applications in AD.

In this study, we first characterized the CNS-tropism of a lab-designed AAVT42 in monkeys and mice. To investigate whether the neuroprotective effects of BDNF are independent of pathogenic protein accumulation, we used AAVT42 to deliver BDNF cDNA to the hippocampus of three distinct AD mouse models: amyloid precursor protein/presenilin-1 (APP/PS1), which exhibits Aβ pathology; rTg4510, which features phosphorylated-tau (p-tau) and neuronal fibrillary tangles (NTFs); and 3 × Tg, which has both Aβ and p-tau accumulation. We assessed the long-term therapeutic effects of BDNF elevation in the hippocampus on learning and memory impairments. Finally, we conducted transcriptomic profiling in the treated hippocampus of 3 × Tg mice to identify differentially expressed genes and pathways associated with neuronal structures, synaptic transmission, and synaptic activities. These analyses revealed potential molecular mechanisms underlying the beneficial effects of BDNF in AD. Our findings may stimulate further research on AAV delivery systems for AD treatment, particularly regarding BDNF-based therapies in clinical trials.

## Materials and methods

### Mice

All procedures complied with the National Research Council Guide for the Care and Use of Laboratory Animals, and all mice were bred in the Shanghai Model Organisms Center. All animal experiments were approved by the Institutional Animal Care and Use Committee (IACUC number: 2023-0002-06). Mice were housed 2–5 per cage under standard conditions, maintained on a 12-h dark and 12-h light cycle with free access to sterile water.

Three strains of AD mice 3 × Tg (Jax strain number 004807), rTg4510 (Jax strain number 007004 and 015815), and APP/PS1 (004462) were purchased from the Jackson Laboratory, USA. As parental gender influences the phenotype of rTg450,^33^ we crossed female tetO-MAPT∗P301L mice (on an FVB/NJ background; 015815) with male CaMKII-tTA mouse line (on a C57BL/6J background; 007004) according to the reference^34^. The Tg4510 and APP/PS1 transgenic groups were identified according to genotype protocols from the suppliers’ instructions. For the experiment design, each group of APP/PS1 and rTg4510 mice had an equal number of females and males. The 3 × Tg group was homozygous for all three mutant alleles, and we did not set the wild-type (WT) control with a similar genetic background and applied females for analysis to reduce gender differences in behavior experiments.

### Monkeys

Two healthy adult male Cynomolgus macaque (Macaca fascicularis) were used. The animals were 11 years old (weight, 8.4 kg) and 14 years old (weight, 6.35 kg) at the start of the experiment. Animal experiments were conducted under the National Institutes of Health guidelines for the care and use of laboratory animals and in accordance with the guidelines approved by the ethics committee for primate research of the Experimental Animal Center of Kunming Medical University, China (IACUC number: kmm120221596).

### AAV vector

A self-complementary AAV (scAAV) construct expressing human cytomegalovirus immediate early enhancer and promoter with the human BDNF cDNA was generated. Viral particles were produced as previously described.^35^ Viruses carrying green fluorescent protein (GFP) or red fluorescent protein (mCherry) were used as controls. For cytomegalovirus-BDNF-ires-mCherry construction, the sequence was inserted into one single-stranded DNA genome.

### Viral injection

The 3−4.5 months transgenic mice were deeply anesthetized with 20 mg/kg commercial Zoletil 50 (Virbac China) mixed with 30 mg/kg Xylazine. After the mouse brain was fixed in the stereotactic apparatus (RWD, Shenzhen, China), the virus was injected bilaterally into the dorsal hippocampus by a micro glass electrode (lab made by Sutter Instrument, inner diameter: 0.58 mm, outer diameter: 1.0 mm) connected to the micro sampler (10 μL, Gaoge, China). Injection site: anteroposterior (A/P) −1.94 mm, lateral (M/L) ± 1.50 mm, ventral(D/V) −1.80 mm. The injection volume was 1 μL at each site, and the viral vector tilter was 4 × 10^12^ genome copies/mL. For intrathecal injection, a total 20 μL of 4 × 10^12^ GC/mL of viruses were injected into L5-vertebrae by micro sampler (25 mL, Gaoge, China).

All monkeys were anesthetized with atropine (0.03–0.05 mg/kg, intramuscularly), and then injected with ketamine (10 mg/kg, intramuscularly) and pentobarbital (Merck, 40 mg/kg, intramuscularly). All surgical procedures were conducted under strict aseptic conditions. As a prophylactic antibiotic treatment, cephalosporin was injected for five consecutive days after surgery (25 mg/kg/day, intramuscularly, once a day). Each monkey's head was fixed on a stereotaxic frame (RWD, Shenzhen, China) and a craniotomy was carried out by dental drilling according to the stereotaxic coordinates obtained from MRI scanning. The AAV virus was then infused through a 31-gauge Hamilton syringe placed in a syringe pump (WPI Apparatus, Sarasota, USA) at a 1 μL/min speed. The viral vector titer was 4 × 10^12^ genome copies/mL, and the total volume was 50 μL. The injection site was aimed at the hippocampus. After infusion, the needle was left in place for 8 min before being slowly retracted from the brain. The same operation was repeated until all injections were finished. After injection, the wound was cleaned thoroughly and sutured.

### Open field test

The locomotor activities of mice were assessed over 30 min in a 60 × 40 cm chamber, in which the center zone was defined as a 36 × 20 cm area. Moving distance, moving duration, and time spent in the center zone were recorded for analysis.

### Y-maze test

A special working memory test was measured following reference^36^. The Y-shaped maze is made of black polyvinyl chloride plastic (20 cm long and 9 cm wide with 20 cm high walls) in which the arms are at 120 angles from each other. Mice were put into the arms of the maze and allowed to explore them. The number of entries and the sequence of entered arms were manually recorded for 8 min. After each test, the arms were thoroughly cleaned with 70 % ethanol to remove residual odors. A spontaneous alternation was defined as entries into three different arms consecutively (*i.e*., a'b’c’, b'c’a’, or c'a’b’, but not a'b’a’ or b'c’b’). The calculation was defined as follows: alternation percentage = (number of alternations)/(total arm entries − 2) × 100.^37^ The number of total arm entries served as an indicator of the locomotor activities.

### Morris water maze

The Morris Water Maze test was conducted in a circular tank (diameter: 125 cm; height: 50 cm; water depth: 30 cm). The water temperature was kept at 20.5−22.5 °C and mice were carried into the testing room every day at least 1 h before the experiment. The whole procedures were refined as reference^38^. The pool was divided into four quadrants, and one escape platform (diameter: 10 cm) was submerged in one quadrant, about 1.5 cm below the water surface. The powder of edible titanium dioxide (JiangHuTaiBai, China) was added to the water to make sure that the platform was not visible. Four direction labels were pasted on the inner wall of the pool above the water, which was the only spatial cue for mice. During each training trial, the animal was put into water for 60 s of swimming. If it found the platform and jumped on it, it would stay here for 12−15 s; or after 60 s, it was guided on the platform and stayed for ∼15 s. Then it was dried and put back to the home cage. A total of four trials were conducted daily with a 30-min interval. The entry position was with a random sequential each day. After 5 days of training, the hidden platform was taken away, then the mice were put into the pool at the position opposite the platform containing the quadrant in the training section for 60-s probe tests, during which the video was recorded using an overhead camera. Results were analyzed with EthoVision® XT 15 software.

### Barnes test

The maze comprises a white circular platform (80 cm in height, 150 cm in diameter) with forty evenly distributed wholes (5 cm in diameter) along its periphery. Beneath one fixed hole, there was a black “escape box”, and mice naturally seeked the escape hole and entered the dark box. Distinct visual cues were displayed on the platform's walls to provide spatial orientation. Before the experiment, each mouse was placed in the escape hole for 1 min. The experiment consists of two parts: training and testing. During training, mice were placed on the platform for 3 min to allow free exploration, if the mouse found the escape hole within 3 min and entered it, the mouse would be then allowed to remain in the escape box for 1 min. The latency of finding the escape hole and the number of errors made before finding the correct hole were recorded. If the mouse did not see the escape hole, the duration for searching the escape box (escape latency) was recorded as 3 min, the mouse was guided to the escape hole and stayed for 1 min. Training was conducted four times daily with a 15−20-min interval. After 4 days of training, most mice quickly found the “escape hole”. On the 12th day, the mice were back to the center of the platform for one more trial, which was regarded as a memory test, and recorded the escape latency.

### Conditioned fear test

The test was conducted in a sound-attenuated chamber with a grid floor capable of delivering an electric shock (Med Associated Inc. USA). Mice movement was recorded and freezing was analyzed with supporting software. On the 1st day, the mice stayed in the chamber for 10 min, and the baseline freezing behavior was recorded. Then after 24 h, an 85-dB white noise presenting 20 s served as the conditioned stimulus (CS). During the final 2 s of the noise, mice received a mild foot shock (0.40 mA), which served as the unconditioned stimulus (US). After 100 s, another CS-US pair was given, with a total of three CS-US pairs stimulus. Tweenty-4 h later, each mouse was taken back into the test chamber, the freezing behavior was recorded for 5 min (context test). Mice were returned to their home cage and placed in another dark room for 2 h. For the conditional cued test, insert an opaque plastic triangle in the chamber and cover the grid floor with the opaque plastic to change the inner shape and spatial cues of the chamber, turn off the white light, wipe the chamber with 30 % isopropyl alcohol instead of 30 % ethanol to alter the smell in the chamber. Then the mouse was placed in the apparatus for 3 min, following continued auditory CS for another 3-min freezing records. Videos were captured and analyzed by the supporting software from Med Associated Inc.

### Western blotting

Mice's hippocampus was dissected and lysed by RIPA buffer (Beyotime, Shanghai, China) with cocktail inhibitors (Bimake, USA). Following quantification, 30 μg of protein from each sample was subjected to SDS-PAGE analysis in Tris-Glycine gel (Beyotime, Shanghai, China). The separated protein was then transferred to a nitrocellulose membrane. The membrane was blocked with 5% milk in TBS-T (Tris-buffered saline-Tween 20) at room temperature for 15 min and incubated with primary antibody: BDNF (Abcam, UK), GFP (Abmart, Shanghai, China), or α-tubulin (Novus Biologicals, USA) overnight at 4 °C. After incubation, it was washed with TBS-T and further incubated with Horseradish peroxidase (HRP) conjugated secondary antibody (Proteintech, Wuhan, China) for 45 min. The membrane was finally washed with TBS-T and developed using the BeyoECL plus kit (Beyotime, Shanghai, China). The blot was imaged by the Chemiluminescence image system (Tanon, Shanghai, China).

### RNA sequencing and analysis

The left half of the mouse hippocampus was dissected in the dish with pre-cold phosphate-buffered saline (PBS) and put into 1 mL TRIzol (Thermo Scientific, USA). Samples were sent to Qian Tang Biotechnology Company (Suzhou, China) in dry ice for RNA extraction (each sample ≥2.0 μg, RIN ≥6.5, 28S:18S ≥ 1.0), library preparation (NEBNext® Ultra™ RNA Library Prep Kit for Illumina), sequencing (Illumina Novaseq), and data analysis.

### Quantitative real-time polymerase chain reaction (qPCR)

RNA was extracted using TRIzol and isopropanol precipitation methods. cDNA was synthesized using cDNA Synthesis Kit (Takara, Japan), qPCR was performed using qPCR SYBR Green Master Mix (Vazyme, China), and results were obtained with a qTOWER3G Real-Time PCR Detection System (Analytik-Jena, Germany). The changes in mRNA levels were calculated using the ΔΔCt method. The primers used are listed in Table 1.

**Table 1:** qPCR primers list.

	Forward (5′-3′)	Reverse (5′-3′)
GAPDH	AGGTCGGTGTGAACGGATTTG	GGGGTCGTTGATGGCAACA
hBDNF	TAACGGCGGCAGACAAAAAGA	TGCACTTGGTCTCGTAGAAGTAT
Npy	ATGCTAGGTAACAAGCGAATGG	TGTCGCAGAGCGGAGTAGTAT
Crh	CCTCAGCCGGTTCTGATCC	GCGGAAAAAGTTAGCCGCAG
Tac1	AAGCGGGATGCTGATTCCTC	TCTTTCGTAGTTCTGCATTGCG
Bmp4	TTGATACCTGAGACCGGGAAG	ACATCTGTAGAAGTGTCGCCTC
Bmp7	ACGGACAGGGCTTCTCCTAC	ATGGTGGTATCGAGGGTGGAA

Note: GAPDH, glyceraldehyde-3-phosphate dehydrogenase; hBDNF: human brain-derived neurotrophic factor; Npy, neuropeptide Y; Crh, corticotropin-releasing hormone; Tac1, tachykinin precursor 1; Bmp4, bone morphogenetic protein 4; Bmp7, bone morphogenetic protein 7.

### Immunohistochemical staining

Mice brains were collected after transcardial perfusion with PBS and 4% paraformaldehyde. Then they were post-fixed in 4% paraformaldehyde for 16 h and cryoprotected sequentially in 10%, 20%, and 30% (wt/vol) sucrose in PBS at 4 °C, embedded in optimal cutting temperature compound for freezing.

Then cryosectioned coronal brain slices (30 μm) were obtained and immunostained for indicated proteins. Primary antibodies were diluted in antibody buffer (containing 0.2% Triton X-100 and 5% bovine serum albumin (BSA)) as follows: anti-Iba1 (1:1000, Wako), anti-NeuN (neuron-specific nuclear protein, 1:2000, Millipore), anti-GFAP (glial fibrillary acidic protein, 1:1000, Millipore), anti-MAP-2 (1:500, Millipore), and anti-Oligo2 (1:500, Millipore) and incubated overnight at 4 °C. Secondary Alexa-conjugated antibodies (Invitrogen) were added at 1:1000 in antibody buffer with DAPI (1:80,000, Millipore) for 2 h at room temperature. Slides were mounted in Vectashield and imaged using the Leica SR5 confocal system. Finally, we used the Leica processing procedure to analyz the fluorescent intensity in the region of interest.

A mouse- and a rabbit-specific HRP/DAB (ABC) detection immunohistochemical staining kit (#ab64264, Abcam, UK) were used for amyloid plaque (4G8, Biolegend), p-tau (AT8, Thermo Fisher) microglia (Iba1, Wako), and astrocyte (GFAP, Millipore) detection by strictly following the manufacturer's instructions, and hematoxylin (Thermo Scientific, USA) was used for counterstaining.

Thirty-nine days after virus injection, monkeys were anesthetized with pentobarbital (45 mg/kg, intramuscularly) and transcardially perfused with 2000 mL of 4 °C PBS and 500 mL of 4% paraformaldehyde (Sigma–Aldrich) in PBS. After perfusion, the hemispheres of the brain were dissected, cut into small blocks, fixed with 4% paraformaldehyde in PBS, and equilibrated in 30% sucrose. The fixed and equilibrated brain tissue blocks were then cut into 50-μm cortical sections with a Microm HM525 cryostat (Microm, Walldorf, Germany). Sections were washed for 5 min in PBS containing 5% BSA and 0.3% Triton X-100, and incubated with primary antibodies (in PBS with 1% BSA and 0.3% Triton X-100) overnight at 4 °C and subsequently with corresponding secondary Alexa-conjugated antibodies (1:1000, Invitrogen). DAPI was used to label the nuclei and sections were mounted with 75% glycerol. Other antibodies used included: anti-GFP antibody (1:1000, Abcam), anti-NeuN (1:2000, Millipore), anti-GFAP (1:800, Biolegend), and anti-Iba1 (1:800, Wako). Confocal z-stack images were acquired on a Nikon A1 confocal laser microscope (Japan).

### Golgi staining and sholl analysis

The 3 × Tg mice were perfused with PBS, and the left hemispheres of the brain were immediately collected and processed according to the FD Rapid Golgi Stain™ kit (FD Neuro Technologies, USA) instructions. Briefly, following pretreatment, brains were sectioned at 100 μm using a cryostat (Leica, Germany) and mounted on gelatin-coated microscope slides for staining. The Leica THUNDER imaging system was used to photograph the neurons in the hippocampus. The branching and length of the dendrites of cornu ammonis 1 (CA1) pyramidal neurons were then analyzed using Image J software and the Simple Neurite tracer (SNT) toolbox, as described in reference^39^.

### Statistical analysis

Data were presented as the mean ± standard error of the mean. The results were analyzed by using Prism 10 software (GraphPad, La Jolla, CA, USA). The significant differences between groups were determined by non-parametric tests, specifically the Mann–Whitney U test for pairwise comparisons or the Kruskal–Wallis test for multiple comparisons. When using parametric tests like *t*-test and ANOVA, we demonstrated the normal distribution of the data through the Shapiro–Wilk test. Significant main effects or interactions were followed up with post-hoc testing using the original false discovery rate method of Benjamini and Hochberg appropriately. The threshold for significance was *P* = 0.05 or false discovery rate = 0.05, with a *P*-value stated in each case. Statistical significance thresholds were set at ^∗^*P* < 0.05, ^∗∗^*P* < 0.01, ^∗∗∗^*P* < 0.001, and ^∗∗∗∗^*P* < 0.0001.

## Results

### AAVT42 achieved efficient neuronal transduction in adult mouse and monkey brains

**Figure 1:**
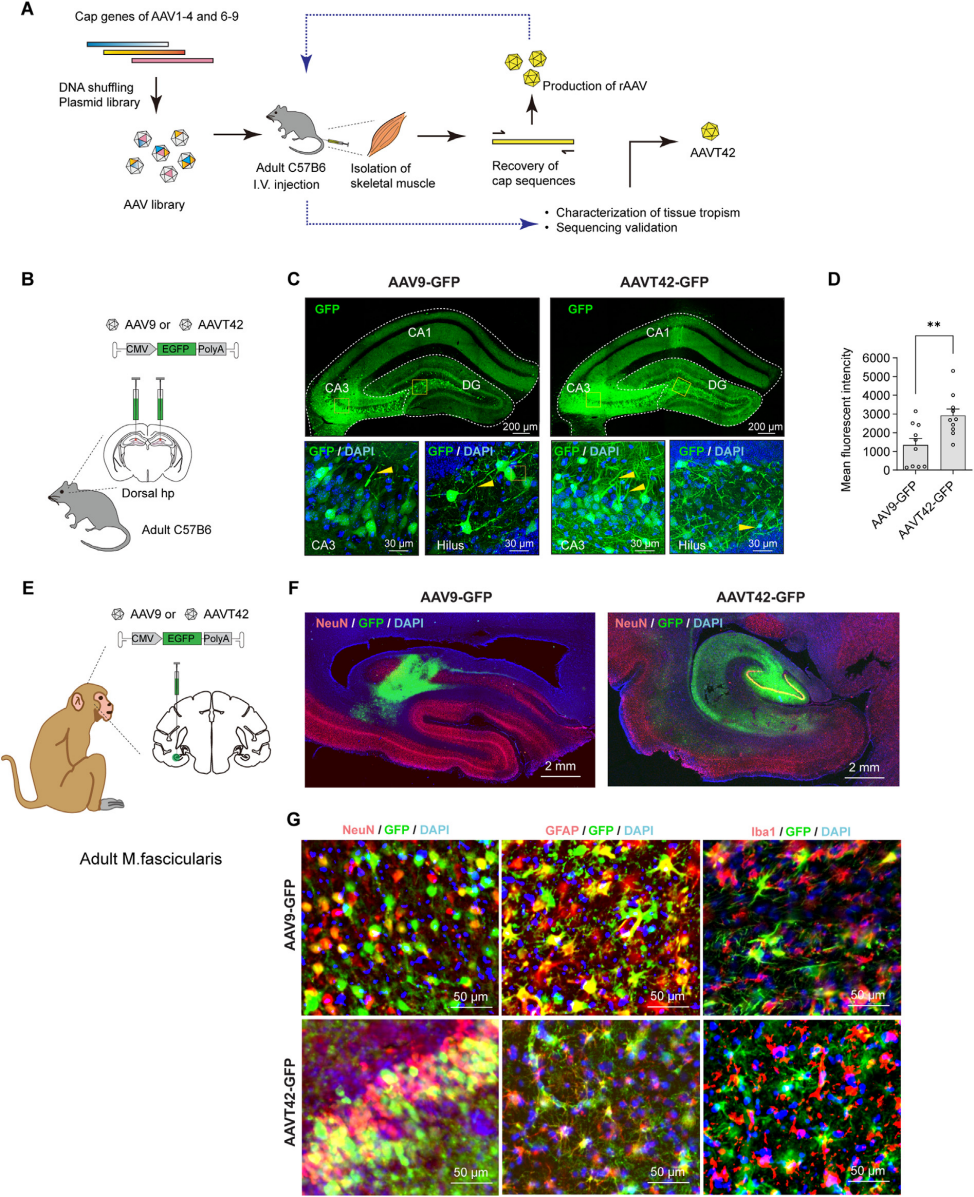
Skeletal muscle targeting AAVT42 vector showed high tropism to the central nervous system in the adult mice and monkeys. **(A)** Generation of the novel AAVT42 vector by directed evolution and *in vivo* screening. The cap genes of AAV1, AAV2, AAV3B, AAV4, and AAV6-9 were mixed, after digestion, denaturation, and reannealing, the shuffled novel AAV cap genes were generated, which were then inserted behind the rep gene and established a library of AAV viral particles. These viruses were injected intravenously in mice, after skeletal muscle isolation, AAV variants displaying tissue tropism were collected, and the corresponding cap genes were recovered by polymerase chain reaction, which were re-cloned into infectious plasmids, forming the viral particles. Then *in vivo* screening in the skeletal muscle was conducted again for further enrichment, characterization, and the sequencing validation of the novel variant. **(B)** The schematic of stereotactic AAV injection in mice. **(C)** Representative confocal microscope images of GFP expression in the mice dorsal hippocampus, and the cell nuclei were marked with DAPI (blue). DG, dentate gyrus; CA1, cornu ammonis 1; CA3, cornu ammonis 3. Yellow arrows indicated GFP-positive dendrites. **(D)** Quantification analysis of GFP fluorescent intensity in the dorsal hippocampus. Both the left and right half of the dorsal hippocampus of each mouse were quantified (dorsal hippocampus: AAV9-GFP = 1342 ± 359, *n* = 5 mice; AAVT42-GFP = 2916 ± 354, *n* = 5 mice, with two injection sites per mouse, AAV9-GFP versus AAVT42-GFP, ∗∗*P* = 6.8 × 10^−3^). **(E)** The schematic of stereotactic AAV injection in monkey brain. **(F)** Confocal images showed GFP-positive cells, which colocalized with neuron marker (NeuN, red) in the hippocampus of monkeys after AAVT42-GFP injection. **(G)** Representative confocal microscope images of antibodies to GFP, NeuN, astrocyte marker GFAP, and microglia marker Iba1 in monkey hippocampus. AAV, adeno-associated virus; GFP, green fluorescent protein.

We used a lab-screened rAAV called AAVT42 for gene delivery into the brain. This viral vector was generated through directed evolution following *in vivo* screening in our lab,^40^ as shown in Figure 1A, which initially targets skeletal muscles. After intrathecal injection of equal genome copies of AAV9-GFP or AAVT42-GFP, stronger GFP signals were observed in the soma of dorsal root ganglia (DRG) transduced with AAVT42-GFP (Fig. S1A–C), indicating that AAVT42 exhibits higher transduction efficiency in the peripheral nervous system. Similarly, when equal genome copies of AAV9-GFP or AAVT42-GFP were injected bilaterally into the mouse dorsal hippocampus (Fig. 1B), the hippocampus transduced with AAVT42-GFP showed higher fluorescent intensity and more clearly labeled cell dendrites, particularly in the Hilus and cornu ammonis 3 (CA3) regions, as indicated by arrows in Figure 1C (signal quantification in Fig. 1D). These results demonstrate that AAVT42 exhibits higher local transduction efficiency in the CNS.

Then we injected viruses in the hippocampus of aged cynomolgus monkeys (Macaca fascicularis*,* over 11 years old), as shown in Figure 1E. Thirty-nine days after injection, GFP fluorescence was detected, indicating efficient gene transfer of GFP. Compared to AAV9-GFP, GFP fluorescence diffused more extensively throughout the hippocampus. Furthermore, the overlap of GFP fluorescence with the neuron-specific nuclear protein NeuN suggested the AAVT42's tropism to neurons (Fig. 1F). To further characterize the GFP-positive cell subtypes, we performed immunostaining with subtype cell markers. The minimal colocalization of GFP fluorescence with astrocyte marker GFAP and microglia marker Iba1 further supported the neuronal specificity of AAVT42 in the brain. In contrast, AAV9 displayed a similar tropism for both neuron and glial cells (Fig. 1G).

**Figure 2:**
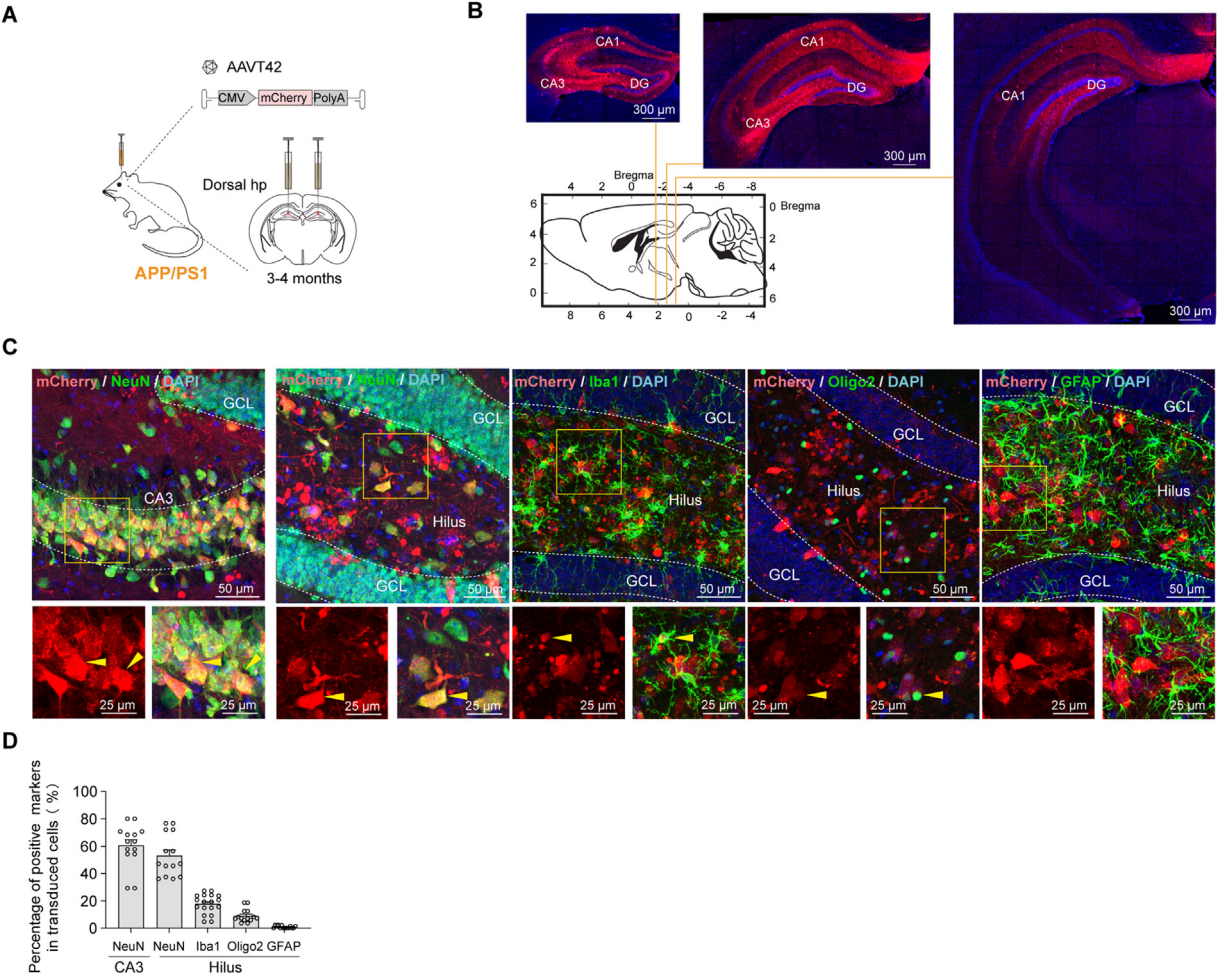
AAVT42 mainly transduced neurons in the adult hippocampus and enabled long-term transgene expression after local injection. **(A)** The schematic of bilateral dorsal-hippo injection of AAVT42-mCherry into 3−4 months APP/PS1 mice, *n* = 3. **(B)** Representative images of mCherry distribution in the hippocampus from dorsal to ventral following the coronal sectioning. Brains were collected when mice were 18–22 months old. **(C)** Representative confocal microscope images of antibodies to mCherry, neuron marker NeuN, astrocyte marker GFAP, microglia marker Iba1, and oligodendrocyte marker Oligo2. An overlapped signal in hippocampal hilus was demonstrated in the lower rectangle and indicated by arrows in yellow. **(D)** Numbers of different markers and their ratio in mCherry-positive cells in the hilus of DG region were calculated. The ratio of NeuN^+^ cells in mCherry^+^ cells in the CA3 region was also quantified. A total of 14–18 pictures of each marker in 3 mice were quantified, and each circle represented a picture from one half coronal hippocampal slice (percentage of NeuN^+^ cells/mCherry^+^ cells in CA1 = 60.54 ± 4.16, in hilus = 53.06 ± 4.13; percentage of Iba1^+^ cells/mCherry^+^ cells in hilus = 17.72 ± 1.68, percentage of Oligo2^+^ cells/mCherry^+^ cells in hilus = 9.02 ± 1.30, percentage of GFAP^+^ cells/mCherry^+^ cells in hilus = 0.83 ± 0.28. DG, dentate gyrus; GCL, granule cell layer.

We then injected AAVT42 carrying the mCherry into the dorsal hippocampus of adult APP/PS1 mice to assess the virus transduction efficiency and identify the cell types in the AD mice (Fig. 2A). Strong mCherry fluorescence signal was observed throughout the dorsal to ventral hippocampus, particularly in the dentate gyrus (DG) and CA3 regions (Fig. 2B), indicating efficient transduction following a single-site local injection. Fluorescent co-labeling of mCherry with the neuronal marker NeuN in the DG and CA1 regions, as well as with the astrocyte marker GFAP, microglial marker Iba1, and oligodendrocyte marker Oligo2 in the DG, is shown in Figure 2C. Quantification of the colocalization of mCherry with these markers (Fig. 2D) revealed that the majority of mCherry-expressing cells were NeuN-positive, suggesting that AAVT42 predominantly transduced neurons, with only a small proportion of microglia and a few astrocytes or oligodendrocytes transduced in adult transgenic mice. Additionally, substantial BDNF expression was detected 4 months after AAVT42-BDNF injection into the hippocampus of 3-month-old C57B6 mice (Fig. S1D, E).

These results collectively demonstrated that AAVT42 is an effective vector for gene delivery to neurons in adult mice. More importantly, it supports long-term transgene expression.

### BDNF overexpression in the hippocampus ameliorated memory impairment in APP/PS1 mice

**Figure 3:**
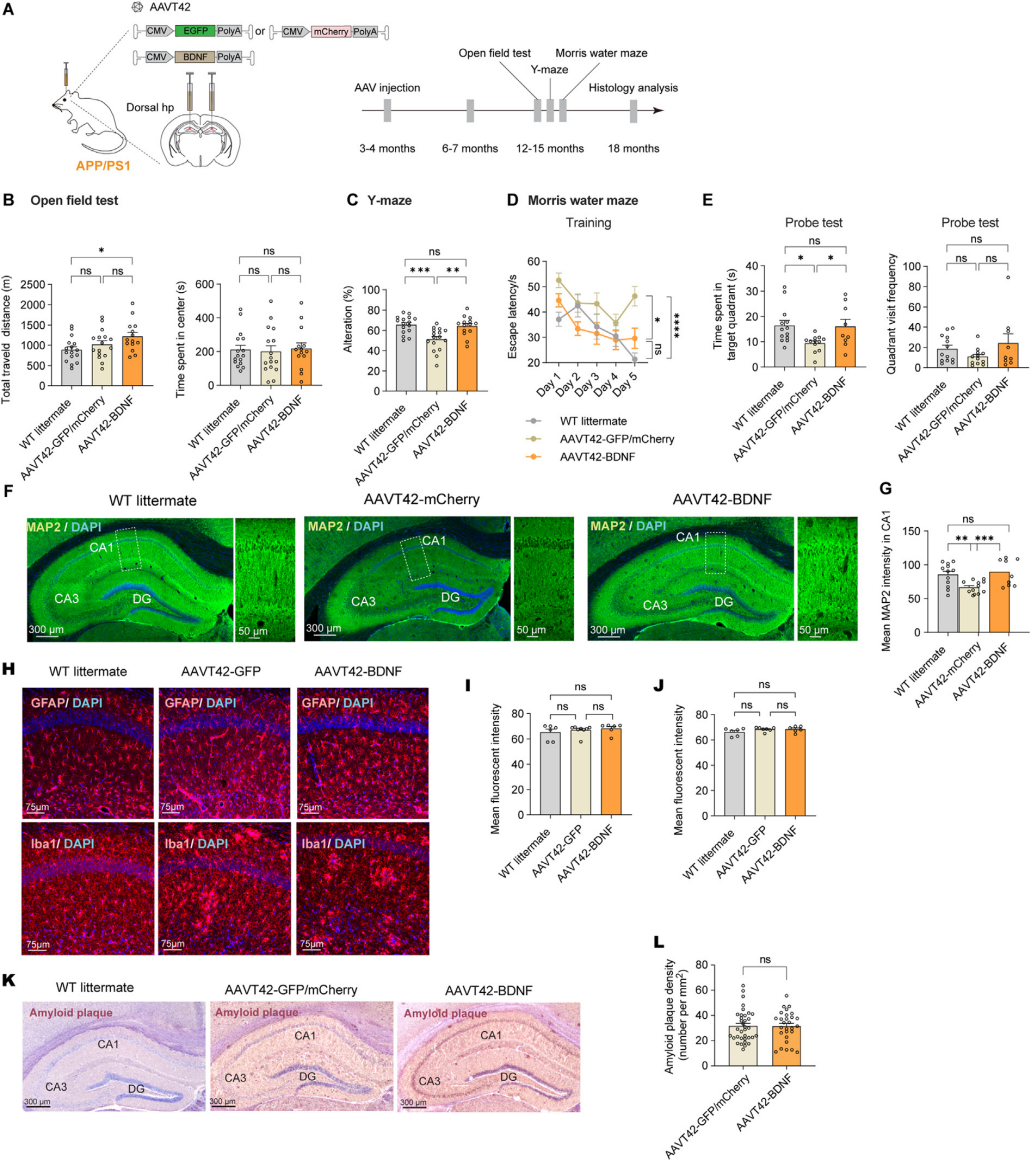
BDNF high expression in hippocampus ameliorated memory impairment in APP/PS1 mice. **(A)** Timeline for experiments in APP/PS1 mice. Wild type (WT) littermate, *n* = 16; AAVT42-GFP/mCherry, *n* = 16; AAVT42-BDNF, *n* = 14. **(B)** Left: The total traveled distance in the open field test. WT littermate: 88.74 ± 7.62 m, AAVT42-GFP/mCherry: 102.10 ± 8.90 m, AAVT42-BDNF: 121.70 ± 10.1 m. *P* = 0.28 for WT littermate *vs.* AAVT42-GFP/mCherry, *P* = 0.13 for AAVT42-GFP/mCherry *vs.* AAVT42-BDNF, ∗*P* = 0.013 for WT littermate *vs.* AAVT42-BDNF. Right: Time spent in the center area. WT littermate: 177.60 ± 16.14 s, AAVT42-GFP/mCherry: 180.50 ± 28.74 s, AAVT42-BDNF: 218.40 ± 33.56 s. **(C)** Percentage of spontaneous alternation in Y maze, and mice age was 12–14 months. WT littermate: 65.50% ± 2.16%, AAVT42-GFP/mCherry: 51.16% ± 2.84%, AAVT42-BDNF: 64.11% ± 2.62%. ∗∗∗*P* = 2.0 × 10^−4^ for WT littermate *vs.* AAVT42-GFP/mCherry, ∗∗*P* = 1.0 × 10^−3^ for AAVT42-GFP/mCherry *vs.* AAVT42-BDNF, *P* = 0.70 for WT littermate *vs.* AAVT42-BDNF. **(D)** Escape latency of cohorts each day during Morris water maze training. On the 5th day, WT littermate: 21.38 ± 2.49 s, *n* = 12, AAVT42-GFP/mCherry: 46.23 ± 3.87 s, *n* = 12, AAVT42-BDNF: 29.61 ± 4.04 s, *n* = 9. ∗∗∗∗*P* < 1.0 × 10^−4^ for WT littermate *vs.* AAVT42-GFP/mCherry, ∗∗*P* = 8.1 × 10^−3^ for AAVT42-GFP/mCherry *vs.* AAVT42-BDNF, *P* = 0.10 for WT littermate *vs.* AAVT42-BDNF. **(E)** Analysis of the probe test on the 6th day. Left: duration in the target quadrant. WT littermate: 16.64 ± 2.16 s; AAVT42-GFP/mCherry: 9.40 ± 0.93 s; AAVT42-BDNF: 16.03 ± 2.78 s ∗*P* = 0.011 for WT littermate *vs.* AAVT42-GFP/mCherry, ∗*P* = 0.029 for AAVT42-GFP/mCherry *vs.* AAVT42-BDNF, *P* = 0.83 for WT littermate vs. AAVT42-BDNF. Right: target quadrant visit frequency. WT littermate: 18.33 ± 3.92 s, AAVT42-GFP/mCherry: 10.83 ± 2.36 s, AAVT42-BDNF: 24.22 ± 9.22 s. *P* = 0.23 for WT littermate *vs.* AAVT42-GFP/mCherry, *P* = 0.27 for AAVT42-GFP/mCherry *vs.* AAVT42-BDNF, *P* > 0.99 for WT littermate *vs.* AAVT42-BDNF. **(F)** Representative images of MAP-2 immunostaining in the hippocampus. The zoomed-in regions were enclosed by the dashed rectangle on the left. **(G)** Mean MAP-2 fluorescence intensity in CA1 region, *n* = 3 for each group, and each mouse quantified four CA1 areas bilaterally. ∗∗*P* = 4.8 × 10^−3^ for WT littermate *vs.* AAVT42-GFP/mCherry, ∗∗∗*P* = 8.0 × 10^−4^ for AAVT42-GFP/mCherry *vs.* AAVT42-BDNF, *P* = 0.51 for WT littermate *vs.* AAVT42-BDNF. **(H−J)** Immunofluorescence of GFAP for astrocytes in CA1 region (quantification of mean fluorescence intensity of GFAP in Figure 3I. WT littermate: 65.18 ± 2.47, *n* = 3; AAVT42-GFP: 66.96 ± 1.32, *n* = 4; AAVT42-BDNF: 68.25 ± 1.66, *n* = 3. Each mouse quantified one brain slice including two CA1 areas bilaterally.) and Iba1 for microglia in CA1 region (quantification of mean fluorescence intensity of Iba1 in Figure 3J. WT littermate: 66.22 ± 1.25, *n* = 3; AAVT42-GFP = 68.38 ± 0.62, *n* = 4; AAVT42-BDNF: 68.51 ± 0.96, *n* = 3. Each mouse quantified one brain slice including two CA1 areas bilaterally). **(K)** Representative images of Aβ (4G8) DAB (3,3′-diaminobenzidine) staining in the hippocampus. **(L)** Quantification of amyloid plaques in the hippocampus. Each mouse quantified four to five brain slices. AAVT42-GFP/mCherry: 31.70 ± 2.14, *n* = 7; AAVT42-BDNF: 31.47 ± 2.28, *n* = 6.

Transgenic APP/PS1 mice, which exhibit amyloid-induced cognitive degeneration, were selected for AD study. AAVT42-BDNF were injected bilaterally into the dorsal hippocampus of 3−4-month-old APP/PS1 mice when pathogenic Aβ accumulation began in the brain. The control group of AD mice received AAVT42-GFP or AAVT42-mCherry injections. Age-matched littermates without transgenes were served as a healthy WT group. Behavioral tests were conducted at 12−15 months of age when neuropathological degeneration and behavioral abnormalities were detectable (Fig. 3A). BDNF treatment did not affect body weight, as shown in Figure S2A. In the open field test, the BDNF-treated mice moved a greater distance (Fig. 3B, left), likely due to increased moving willingness resulting from increased BDNF levels in the hippocampus. This was further supported by the increased velocity observed after BDNF treatment (Fig. S2B). No significant difference was observed in the time spent in the center zone among the three groups (Fig. 3B, right), indicating that APP/PS1 animals did not exhibit anxiety-like behaviors.

Next, hippocampus-dependent memory performance was assessed. Mice with severe hair loss and injuries from fighting were excluded from the experiment. In the Y-maze test, AAVT42-GFP/mCherry injected mice showed a reduced spontaneous alternation ratio compared to WT littermates, indicating impaired spatial working memory. AAVT42-BDNF treatment alleviated this deficit (Fig. 3C). In the Morris water maze, the escape latency of WT littermates and AAVT42-BDNF-treated mice increased over five consecutive training days, suggesting efficient learning ability. However, the escape latency of the APP/SP1 AAVT42-GFP/mCherry mice remained nearly stable, implying impaired learning ability (Fig. 3D). Consistently, the BDNF-treated group spent more time in the target quadrant during the probe test, demonstrating improved spatial memory retention (Fig. 3E, left panel). All groups showed similar quadrant visit frequency (Fig. 3E, right panel). These results from both behavioral paradigms consistently indicated that BDNF overexpression in the dorsal hippocampus rescued memory deficits in APP/PS1 mouse.

Microtubule-associated protein-2 (MAP-2) is a dendritic marker crucial for neuronal development, differentiation, plasticity, and neurogenesis in the hippocampus by maintaining neural structure.^41^^,^^42^ Fluorescent staining revealed decreased MAP-2 intensity in the CA1 region of AD transgenic mice compared with WT littermates, which was restored following BDNF treatment (Fig. 3F, G), indicating enhanced neuronal structure. To assess the immune response to BDNF overexpression, the intensities of astrocytes (labeled with GFAP) and microglia (labeled with Iba1) in the CA1 region were measured. The results showed no obvious effect of BDNF injection on either astrocytes or the microglia populations in the hippocampus in Figure 3H, the corresponding signal intensities in the CA1 region were quantified in Figure 3I and J. Finally, DAB (3,3′-diaminobenzidine) staining revealed no significant difference in the number of amyloid plaques in the hippocampus between the treatment and control groups (Fig. 3K, L).

Overall, APP/PS1 model demonstrates that AAVT42-mediated delivery of BDNF into the hippocampus promotes neuroprotection against Aβ-induced neurotoxicity and strengthens dendritic structure during AD pathogenesis, thus alleviating cognitive impairment.

### High BDNF expression in the hippocampus ameliorated memory impairment in rTg4510 and 3 × Tg mice

**Figure 4:**
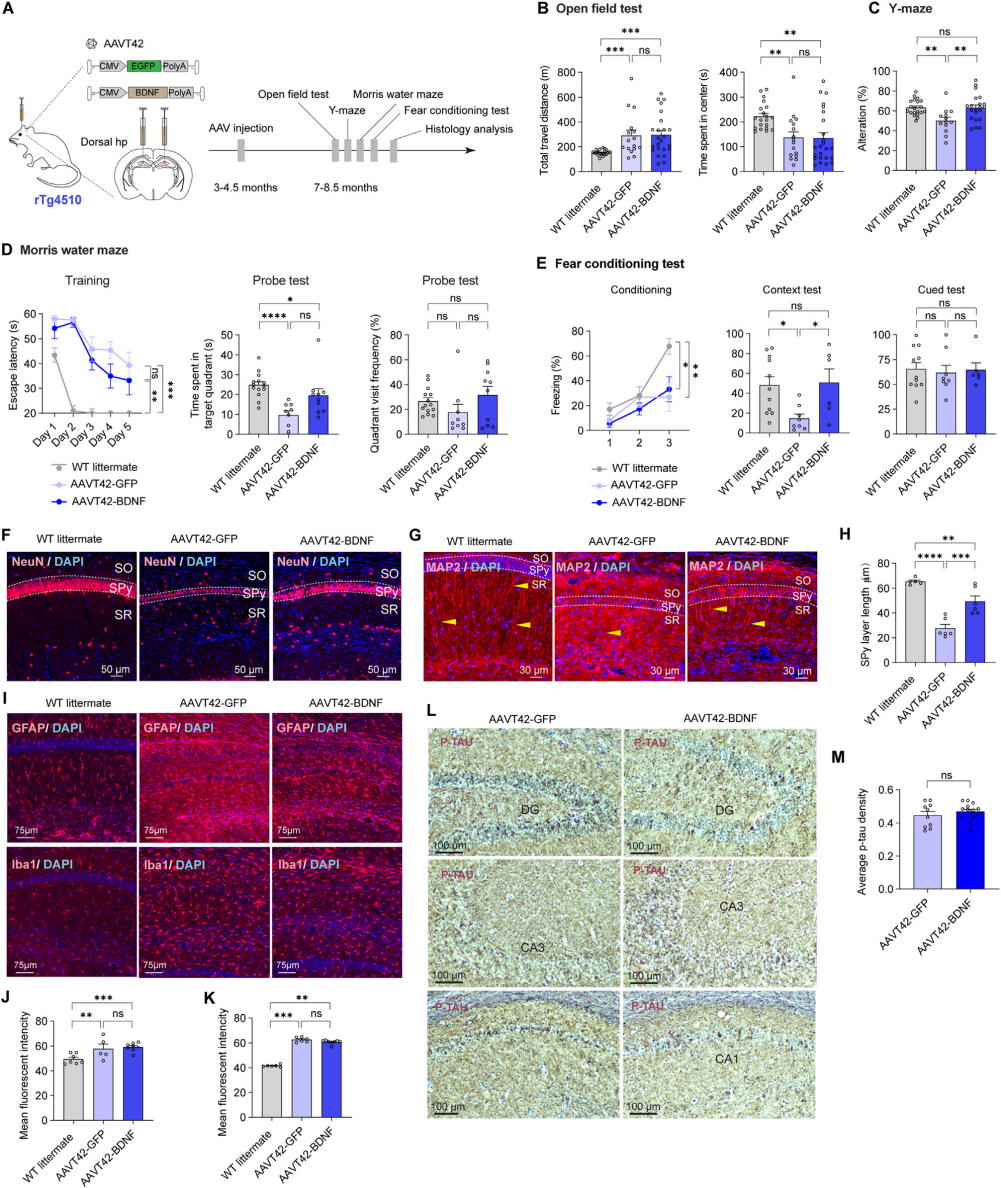
BDNF high expression in hippocampus ameliorated memory impairment in rTg4510 mice. **(A)** Timeline for experiments in rTg4510 mice. WT littermate, *n* = 20; AAVT42-GFP, *n* = 16; AAVT42-BDNF, *n* = 24. **(B)** Analysis of open field test, age was 7–8 months. Left: total traveled distance. WT littermate: 152.6 ± 4.9 m; AAVT42-GFP: 291.1 ± 43.6 m; AAVT42-BDNF: 296.5 ± 34.0 m ∗∗∗*P* = 8.0 × 10^−4^ for WT littermate *vs.* AAVT42-GFP, *P* = 0.93 for AAVT42-GFP *vs.* AAVT42-BDNF, ∗∗∗*P* = 1.0 × 10^−4^ for WT littermate *vs.* AAVT42-BDNF. Left: time spent in the center area. WT littermate: 222.4 ± 12.05 s; AAVT42-GFP:136.7 ± 22.85 s; AAVT42-BDNF:134.8 ± 21.54 s ∗∗*P* = 4.3 × 10^−3^ for WT littermate *vs.* AAVT42-GFP, *P* = 0.91 for AAVT42-GFP *vs.* AAVT42-BDNF, ∗∗*P* = 1.0 × 10^−3^ for WT littermate *vs.* AAVT42-BDNF **(C)** Percentage of spontaneous alternation in Y maze. WT littermate, *n* = 20; AAVT42-GFP, *n* = 13; AAVT42-BDNF, *n* = 21. WT littermate: 63.24% ± 1.77%; AAVT42-GFP: 50.10% ± 3.49%; AAVT42-BDNF: 62.93% ± 2.93%. ∗∗*P* = 2.2 × 10^−3^ for WT littermate *vs.* AAVT42-GFP, ∗∗*P* = 2.6 × 10^−3^ for AAVT42-GFP *vs.* AAVT42-BDNF, *P* = 0.93 for WT littermate *vs.* AAVT42-BDNF **(D)** Analysis of Morris water maze. Escape latency of cohorts each day during Morris water maze training. WT littermate, *n* = 14; AAVT42-GFP, *n* = 9; AAVT42-BDNF, *n* = 10. On the 5th day, WT littermate: 11.70 ± 1.53 s; AAVT42-GFP: 39.39 ± 5.11 s; AAVT42-BDNF: 33.18 ± 5.78 s ∗∗∗*P* = 5.0 × 10^−4^ for WT littermate *vs.* AAVT42-GFP, *P* = 0.43 for AAVT42-GFP *vs.* AAVT42-BDNF, ∗∗*P* = 4.7 × 10^−3^ for WT littermate *vs.* AAVT42-BDNF. For probe tests, quantification of time spent in the target quadrant zone (WT littermate: 24.73 ± 1.70 s; AAVT42-GFP: 9.66 ± 2.09 s; AAVT42-BDNF: 19.51 ± 3.42 s ∗∗∗∗*P* < 1.0 × 10^−4^ for WT littermate *vs.* AAVT42-GFP, *P* = 0.066 for AAVT42-GFP *vs.* AAVT42-BDNF, ∗*P* = 0.033 for WT littermate vs. AAVT42-BDNF) and target quadrant visit frequency (WT littermate: 26.50 ± 2.82; AAVT42-GFP: 17.56 ± 6.62; AAVT42-BDNF: 31.60 ± 6.94) **(E)** Percentage of freezing time during three consecutive stimuli. At 3rd stimulus, freezing ratio of WT littermate: 67.674 ± 6.397 %, *n* = 11; AAVT42-GFP = 26.744% ± 11.528%, *n* = 8; AAVT42-BDNF = 33.092% ± 10.068%, *n* = 6. ∗∗*P* = 9.0 × 10^−3^ for WT littermate *vs.* AAVT42-GFP, *P* = 0.69 for AAVT42-GFP *vs.* AAVT42-BDNF, ∗*P* = 0.017 for WT littermate *vs.* AAVT42-BDNF. The freezing ratio during contextual test: WT littermate, 48.02% ± 8.57%; AAVT42-GFP, 14.75% ± 4.38%; AAVT42-BDNF, 50.64% ± 13.71%. ∗*P* = 0.011 for WT littermate *vs.* AAVT42-GFP, ∗*P* = 0.018 for AAVT42-GFP *vs.* AAVT42-BDNF, *P* = 0.84 for WT littermate *vs.* AAVT42-BDNF. The freezing ratio during the conditioned-cue test: WT littermate, 65.46% ± 6.43%; AAVT42-GFP, 61.67% ± 7.56%; AAVT42-BDNF, 64.53% ± 7.03% **(F, G)** Representative images of NeuN and MAP-2 immunostaining in the CA1 region of the hippocampus, age was around 12 months **(H)** Quantification of the thickness of SPy layer of NeuN fluorescence in CA1 region. *n* = 3 for each group. WT littermate: 65.40 ± 1.15 μm; AAVT42-GFP, 27.70 ± 3.12 μm; AAVT42-BDNF, 49.40 ± 4.36 μm ∗∗∗∗*P* < 1.0 × 10^−4^ WT littermate *vs.* AAVT42-GFP, ∗∗∗*P* = 3.0 × 10^−4^ AAVT42-GFP *vs.* AAVT42-BDNF, ∗∗*P* = 5.1 × 10^−3^ for WT littermate *vs.* AAVT42-BDNF. Each point represents one half of the coronal hippocampus **(I–K)** Immunofluorescence of GFAP for astrocytes in CA1 region (quantification of mean fluorescence intensity in Figure 4J. WT littermate: 47.47 ± 0.87, *n* = 3; AAVT42-GFP: 57.61 ± 3.86, *n* = 3; AAVT42-BDNF: 59.05 ± 1.19, *n* = 4. ∗∗*P* = 4.1 × 10^−3^ for WT littermate *vs.* AAVT42-GFP, *P* = 0.62 for AAVT42-GFP *vs.* AAVT42-BDNF, ∗∗∗*P* = 6.0 × 10^−4^ for WT littermate *vs.* AAVT42-BDNF), and Iba1 for microglia in CA1 region (quantification of mean fluorescence intensity in Figure 4K. WT littermate: 41.56 ± 0.36, *n* = 3; AAVT42-GFP: 62.59 ± 0.79, *n* = 3; AAVT42-BDNF: 60.69 ± 0.59, *n* = 4. ∗∗∗*P* = 4.0 × 10^−4^ for WT littermate *vs.* AAVT42-GFP, *P* = 0.27 for AAVT42-GFP *vs.* AAVT42-BDNF, ∗∗*P* = 7.8 × 10^−3^ for WT littermate *vs.* AAVT42-BDNF) **(L)** Representative images of phosphorylated Tau (AT8) DAB staining in the hippocampus **(M)** Quantification of phosphorylated Tau in the hippocampus, mice age of 9–12 months. AAVT42-GFP: 0.45 ± 0.023, *n* = 5; AAVT42-BDNF: 0.48 ± 0.014, *n* = 7. Each mouse was quantified in dentate gyrus and CA3 regions. SO, stratum oriens; SR, stratum radiatum; SPy, stratum pyramidale.

Numerous studies have linked neuronal loss and dysfunction to tau hyperphosphorylation, which promotes the formation of neurofibrillary tangles.^43^ We selected rTg4510 mice for further investigation as phosphorylated tau was widely distributed in the frontal cortex and hippocampus. Littermates that did not carry transgenes were regarded as WT littermate control. rAAV was injected into the mice at 3–4.5 months of age, and behavioral deficits were assessed at approximately 6.5–7 months of age (Fig. 4A). As previously reported,^44^^,^^45^ bi-transgenic mice exhibited reduced body weights and hippocampal injection of AAVT42-BDNF did not change this condition (Fig. S3A).

Mice with injuries, such as anal prolapse, were excluded from the behavioral experiments. In the open field test, rTg4510 mice injected with AAVT42-GFP exhibited hyperactivity, as a greater distance traveled shown in Figure 4B (left panel), consistent with previously reported characteristics of rTg4510 mice.^46^ Increased velocity was observed after BDNF treatment (Fig. S3B), similar to the findings in the APP/PS1 model. However, rTg4510 mice spent less time in the center area compared to WT littermates, indicating higher anxiety levels, and BDNF treatment had no significant change in this behavior (Fig. 4B, right panel).

In the Y-Maze test, the reduced alteration percentage in the AAVT42-GFP control group demonstrated memory deficits, which were alleviated by BDNF treatment (Fig. 4C). No significant improvement was observed following BDNF treatment in the Morris water maze test (Fig. 4D). During the conditioned fear test training period, an increasing freezing response was observed in all groups, indicating a learning curve (Fig. 4E, left panel). In the context test, mice treated with BDNF exhibited a higher freezing ratio than GFP-control littermates (Fig. 4E, middle panel), demonstrating improved memory for associating the environment with unpleasant memory. In the following conditioned cue test, all groups of mice responded to the cue stimulus, suggesting that they all had the association of the adverse noise with electric shock in mind (Fig. 4E, right panel).

Previous reports have shown that ventricle injection of AAV8-BDNF rescues neuronal loss in the CA1 region of the rTg4510 mice hippocampus.^47^ Consistent with their results, our study found a broader stratum pyramidal (Spy) layer in the CA1 region after BDNF administration, indicating prevention of neuronal loss (Fig. 4F, the width of the Spy layer was quantified in Fig. 4H). Consistent with compromised white matter integrity in rTg4510 mice,^48^ we also observed disrupted MAP-2 signals in the CA1 region, indicating aberrant dendritic structures (shown by arrows in Fig. 4G). In the BDNF-treated group, an organized MAP-2 pattern similar to that in WT littermates suggested the preservation of dendritic structure.

Increased GFAP and Iba1 signals in the CA1 region of the AAV42-GFP group indicated the active astrogliosis and microglial activation in rTg4510 (Fig. 4I–K), as previously reported,^49^^,^^50^ on which BDNF treatment showed no obvious effect. Finally, DAB staining of AT8 showed that BDNF overexpression did not affect tau phosphorylation in the hippocampus (Fig. 4L, M).

**Figure 5:**
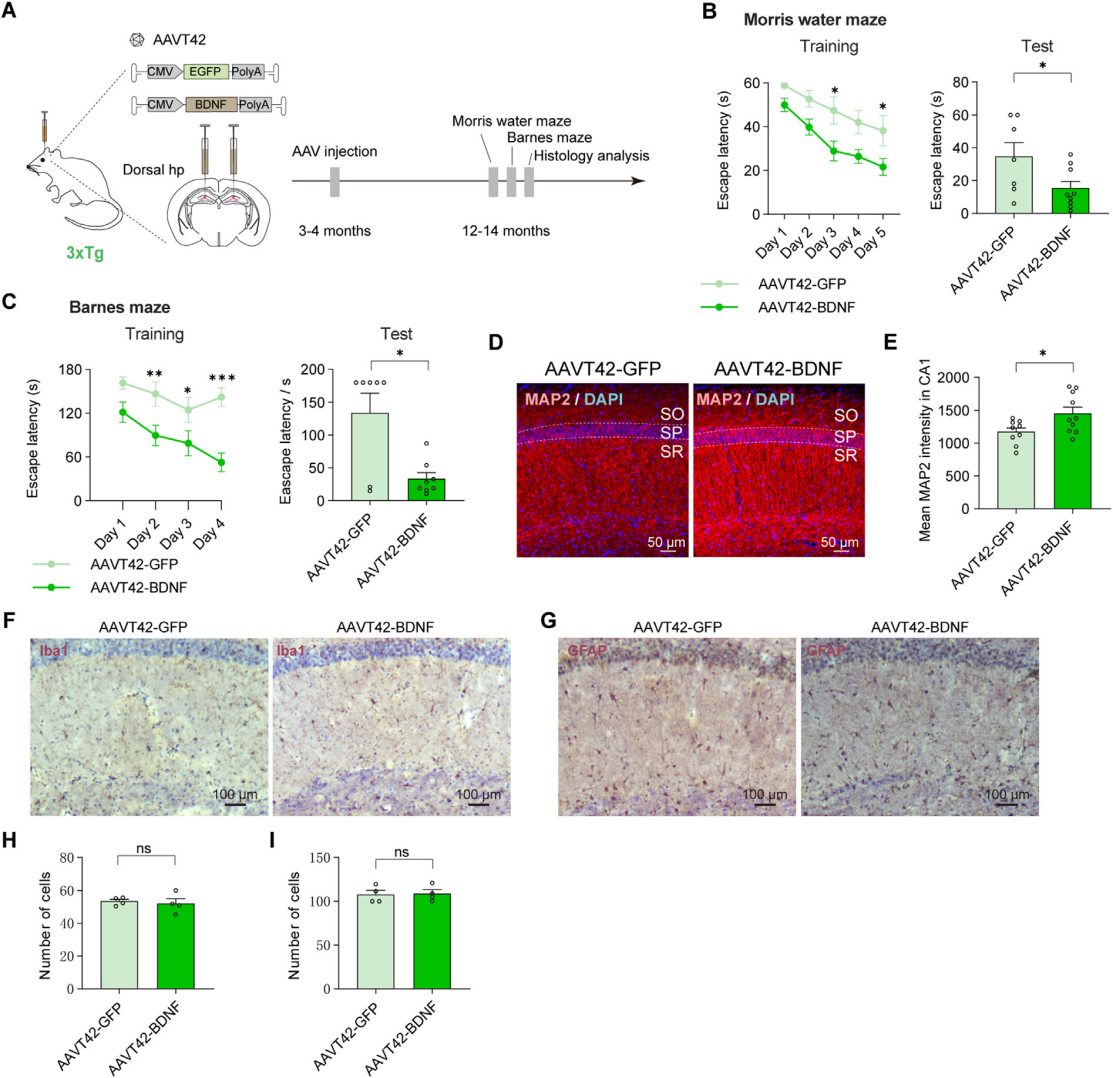
BDNF high expression in hippocampus ameliorated memory impairment in 3 × Tg mice. **(A)** Timeline for experiments in 3 × Tg mice. **(B)** Analysis of Morris water maze. On the 5th day, AAVT42-GFP: 38.17 ± 6.95 s, *n* = 7; AAVT42-BDNF: 21.69 ± 3.82 s, *n* = 9. ∗*P* = 0.044; On the 3rd day, ∗*P* = 0.017. A test on the 6th day was analyzed, AAVT42-GFP: 34.72 ± 8.50 s; AAVT42-BDNF: 15.43 ± 4.16 s ∗*P* = 0.046. **(C)** Analysis of Barnes maze. On the 4th day, AAVT42-GFP: 142.07 ± 12.84 s, *n* = 7; AAVT42-BDNF: 52.75 ± 12.98 s, *n* = 8. ∗∗∗*P* < 0.0001; On the 3rd day, ∗*P* = 0.03; On the 2nd day, ∗∗*P* = 0.008. A test on the 11th day was analyzed. AAVT42-GFP: 133.70 ± 29.88 s; AAVT42-BDNF: 33.38 ± 9.01 s ∗*P* = 0.046. **(D)** Representative images of MAP-2 immunostaining in the CA1 region of the hippocampus, age = 12 months. **(E)** Quantification of mean MAP-2 fluorescence intensity in the CA1 region of the hippocampus. AAVT42-GFP: 1170 ± 60.29, *n* = 5; AAVT42-BDNF: 1452 ± 94.48, *n* = 6. AAVT42-GFP *vs.* AAVT42-BDNF, ∗*P* = 0.0252. Each mouse was quantified one to two CA1 regions. **(F, G)** Representative images of microglia (Iba1) and astrocyte (GFAP) DAB staining in the CA1 region. **(H, I)** Quantification of microglia (AAVT42-GFP: 53.4 ± 1.20, *n* = 4; AAVT42-BDNF: 52.02 ± 3.02, *n* = 4. *P* = 0.687) and astrocytes (AAVT42-GFP: 107.9 ± 4.79, *n* = 4; AAVT42-BDNF: 108.9 ± 4.41, *n* = 4. *P* = 0.884). Each point represents the average cell count in the CA1 region of each mouse based on three to four slide quantifications.

We further investigated the therapeutic effect of BDNF in 3 × Tg mice, which exhibit the accumulation of both Aβ and p-tau in the cortex and hippocampus. AAV vectors were injected into 3−month-old adult mice, and their performance was assessed at 12−14 months of age (Fig. 5A). Consistent with the results observed in APP/PS1 mice, the BDNF-treated group outperformed the control group during five consecutive days of Morris water maze training (Fig. 5B, left panel). In a subsequent test conducted on the 6th day, BDNF-treated mice spent less time searching for the hidden platform compared to controls (Fig. 5B, right panel), indicating improved learning ability and memory. Additionally, the Barnes maze test demonstrated a similar therapeutic effect of BDNF (Fig. 5C). Immunostaining showed enhanced MAP-2 signals in the CA1 region of the hippocampus in 3 × Tg mice (Fig. 5D, E), a pattern similar to that seen in APP/PS1 and rTg4510 mice. Golgi staining and Sholl analysis showed enhanced dendritic complexity in BDNF-treated pyramidal neurons (Fig. S4A, B), further supporting the results that increased local expression of BDNF maintains the dendrite structure, potentially alleviating the neurotoxicity associated with Aβ and p-tau accumulation.

There was no significant change in the number of astrocytes or microglia in the CA1 region, further confirming that BDNF overexpression did not broadly influence immune activation (Fig. 5F–I). Finally, DAB staining of amyloid plaque and phosphorylated Tau in 3 × Tg mice revealed no significant change following BDNF administration (Fig. S4C, D).

### RNA-sequencing analysis identified genes and pathways maintaining neuron structure, mediating synaptic plasticity, and memory formation after long-term BDNF supplementation in neurons

**Figure 6:**
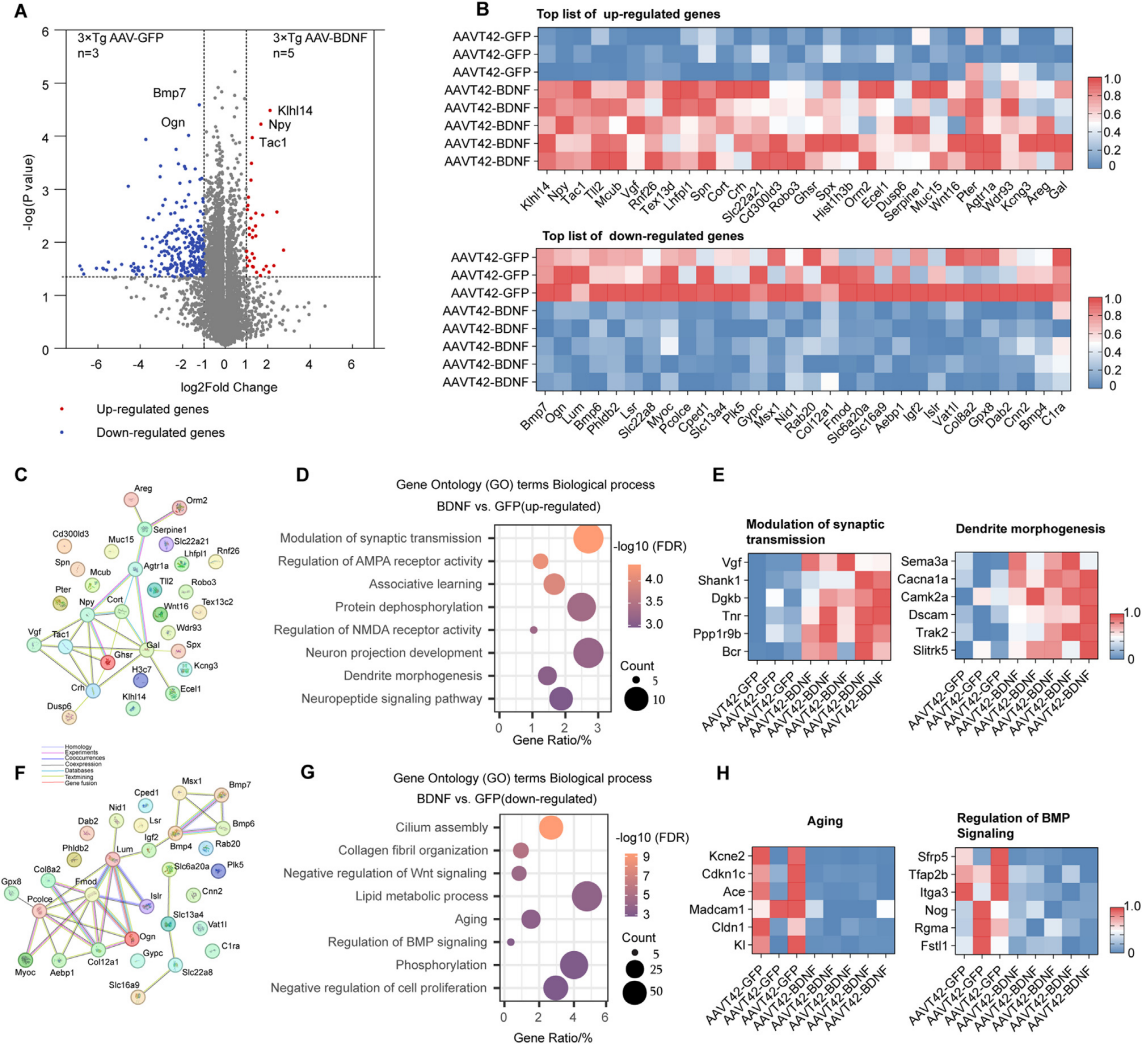
AAVT42-BDNF treatment mainly regulated pathways mediating neuronal structure organization and synaptic transmission in 3 × Tg mice. **(A)** The volcano plot illustrating differentially expressed genes (DEGs) in the AAVT42-BDNF treatment and AAVT42-GFP hippocampus. Dotted lines highlighting the *P*-value = 0.05 (y-axis) and fold change = 2 (x-axis), blue dots showed genes expressed at higher levels in AAVT42-GFP hippocampus, and red dots showed genes expressed at higher levels in AAVT42-BDNF hippocampus. **(B)** The expression heat map of TOP 30 up-regulated genes and down-regulated genes of AAVT42-BDNF *vs.* AAVT42-GFP. **(C)** STRING protein–protein interaction work. Network of top 30 up-regulated proteins in the hippocampus after AAVT42-BDNF administration. Different colors reflected different kinds of connection evidence between proteins. **(D)** Gene ontology enrichment analysis of the up-regulated DEGs for biological process. **(E)** The expression heat map of DEGs enriched in the modulation of synaptic transmission and dendrite morphogenesis. **(F)** STRING protein–protein interaction work. Network of top 30 down-regulated proteins in the hippocampus after AAVT42-BDNF administration. Different colors reflected different kinds of connection evidence between proteins. **(G)** Gene ontology enrichment analysis of the down-regulated DEGs for biological process. **(H)** The expression heat map of DEGs enriched in the Aging and Regulation of BMP signaling.

RNA sequencing was performed to obtain the hippocampus transcriptome from 3 × Tg mice treated with either AAVT42-GFP or AAVT42-BDNF, aiming to identify principal genes and related pathways involved in the cognitive improvement observed in AD. A volcano plot was generated to visualize the expression of 14,678 genes across the two groups, revealing 461 up-regulated genes and 1724 down-regulated genes. Among these, 33 up-regulated and 119 down-regulated differentially expressed genes were identified with a fold change greater than 2 (|log2(fold change)| ≥ 1) (Fig. 6A). The expression patterns of the top 30 up- and down-regulated genes are shown as heat maps in Figure 6B, and STRING analysis of these genes is presented in Figure 6C–F.

Gene ontology analysis of the data indicated that the 461 up-regulated genes were enriched in biological processes related to synaptic structures and functions, such as synaptic transmission modulation, regulation of receptor activity, dendrite morphogenesis, and the neuropeptide signaling pathway (Fig. 6D). Notably, genes involved in synaptic transmission, including the neurosecretory protein *Vgf* and the scaffold protein *Shank1*, were up-regulated following BDNF treatment, suggesting the therapeutic effects of BDNF in AD.^51^^,^^52^ Furthermore, genes involved in dendritic development, such as *Sema3a*,^53^ *Dscam*,^54^ and *Slitrk5*^55^ were also up-regulated, indicating that dendrite structure maintenance was strengthened following BDNF overexpression (Fig. 6E).

In contrast, 1724 down-regulated genes were found to be involved in cellular structural organization, aging, lipid metabolism, negative regulations of Wnt, and BMP signaling pathways (Fig. 6G). The down-regulated genes associated with aging and AD pathogenesis, such as *Kcne2*,^56^^,^^57^ *Cdkn1c*,^58^ *Ace*,^59^ and *Madcam1*,^60^ as well as genes related to the down-regulation of the BMP signaling pathway,^61^ highlighting the therapeutic effects of local BDNF overexpression (Fig. 6H).

### Gene expression was further confirmed in 3 × Tg hippocampus after AAVT42-BDNF administration

**Figure 7:**
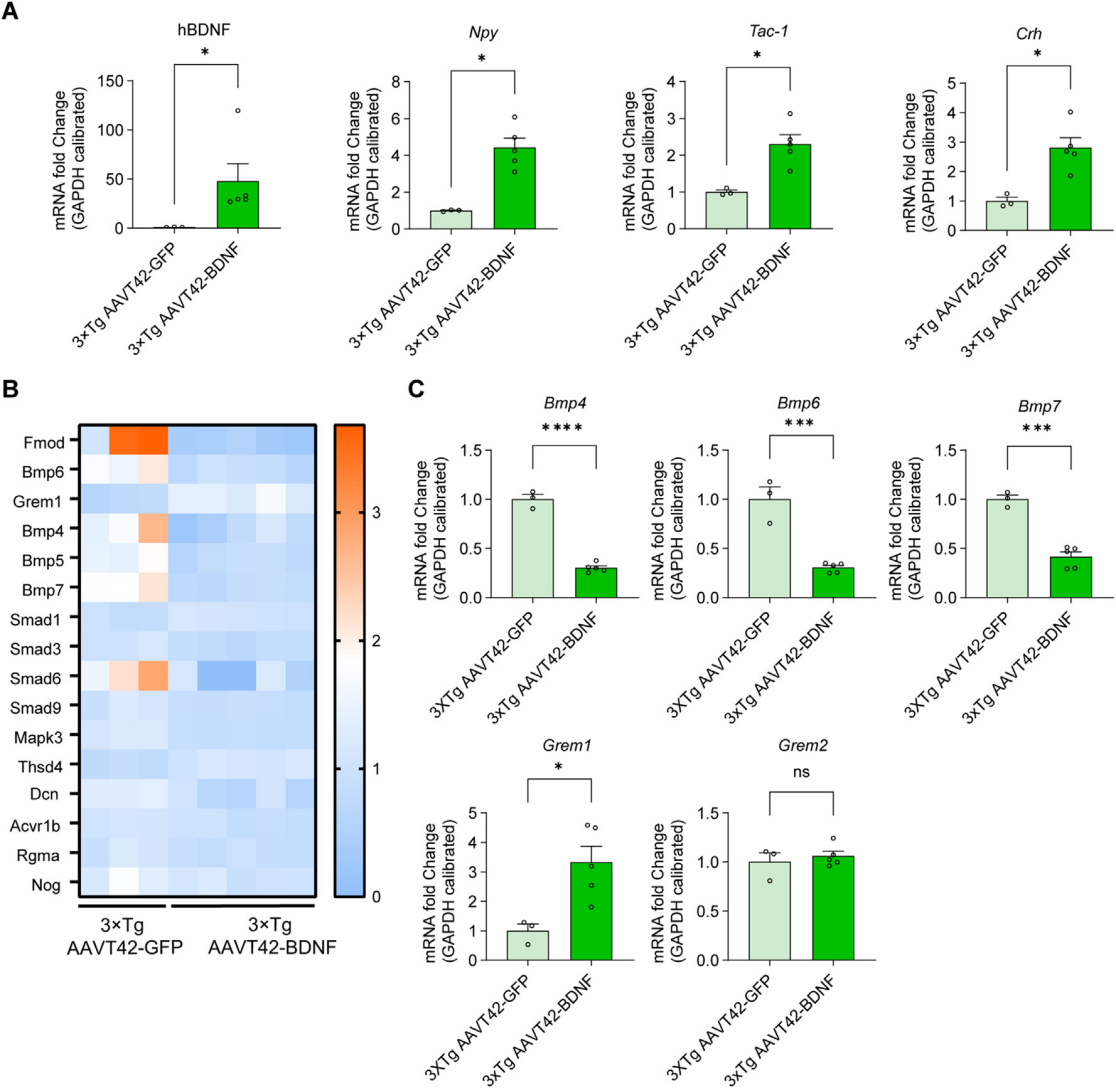
Regulated gene expression levels after AAVT42-BDNF administration were further confirmed in 3 × Tg mice. **(A)** Confirmation of the up-regulated BDNF, *Npy, Tac1, and Crh* expression in the hippocampus by real-time qPCR. All gene expressions in AAVT42-GFP were normalized to one. BDNF: AAVT42-GFP = 1.00 ± 0.11, *n* = 3; AAVT42-BDNF = 47.79 ± 18.01, *n* = 5. *Npy*: AAVT42-GFP = 1.00 ± 0.025; AAVT42-BDNF = 4.42 ± 0.52. *Tac1*:AAVT42-GFP = 1.00 ± 0.054; AAVT42-BDNF = 2.30 ± 0.25. *Crh*: AAVT42-GFP = 0.52 ± 0.068; AAVT42-BDNF = 1.47 ± 0.18. ∗*P* = 0.036. **(B)** The heat map of DEGs in the TGFβ/BMP signaling pathway in 3 × Tg hippocampus after BDNF administration. **(C)** Genes involved in the BMP signaling pathway were detected by real-time qPCR. AAVT42-GFP *vs.* AAVT42-BDNF, *Bmp4*: AAVT42-GFP = 1.00 ± 0.05; AAVT42-BDNF = 0.30 ± 0.02. ∗∗∗∗*P* < 1.0 × 10^−4^; *Bmp6*: AAVT42-GFP = 1.00 ± 0.17; AAVT42-BDNF = 0.31 ± 0.021. ∗∗∗*P* = 0.0004; *Bmp7*: AAVT42-GFP = 1.00 ± 0.044; AAVT42-BDNF = 0.42 ± 0.049. ∗∗∗*P* = 0.0002; *Grem1*: AAVT42-GFP = 1.00 ± 0.23; AAVT42-BDNF = 3.33 ± 0.24. ∗*P* = 0.021; *Grem2*: AAVT42-GFP = 1.00 ± 0.055; AAVT42-BDNF = 1.06 ± 0.049. ∗*P* = 0.55. *Npy*, Neuropeptide Y; *Crh*, Corticotropin-releasing hormone; *Tac1*, Tachykinin precursor 1; *Bmps*, Bone morphogenetic proteins, *Grem1*, Gremlin 1.

Finally, we confirmed the expression of BDNF in 3 × Tg hippocampus, as well as the up-regulated genes such as *Npy*, *Tac1*, and *Crh*, as shown in Figure 7A. Interestingly, Npy is a secreted neurotransmitter or neuromodulator that performs a variety of roles in neurodegenerative diseases, including neuroprotection, increased trophic support, decreased excitotoxicity, calcium homeostasis regulation, and neuroinflammation attenuation.^62^ *Crh* protects neurons against AD pathogenesis,^63^ and *Tac1* serves as a potential vasodilator and increases blood flow based on neural impulses. *Tac1* was recently identified as a hub gene connected to synaptic function and inflammation. All of these genes can be regulated by BDNF overexpression.^64^, ^65^, ^66^

Previous studies have found that *Bmp4* and *Bmp6* are abnormally elevated in AD mice and AD patients.^67^^,^^68^ In contrast, antidepressants can down-regulate BMP signaling, stimulate local neurogenesis, and improve learning ability and memory in AD patients,^69^ besides, enhancing neurogenesis can elicit anti-stress or anti-depression effects.^70^ These studies suggested a negative effect of neurogenesis on BMP signaling. Our RNA sequencing data revealed the decreased expression of genes mediating the TGF-β/BMP signaling pathway in 3 × Tg mice, indicating the inhibitory effect of BDNF overexpression on BMP signaling (Fig. 7B). The down-regulated *Bmp4*, *Bmp6*, and *Bmp7* were further confirmed by qPCR test (Fig. 7C). Consistently, *Grem1*, a negative regulator of TGF-β/BMP signaling^71^ was up-regulated, while *Grem2* expression had no significant change (Fig. 7C).

Our findings confirmed that owing to multi-downstream targets of BDNF, its overexpression will activate multiple downstream signaling pathways, eventually resulting in synergistic neuroprotective functions against neuron degeneration in AD.

## Discussion

In this study, we utilized rAAV vectors to increase local BDNF levels in hippocampal neurons through direct intraparenchymal injection in the early adult stages of three strains of transgenic AD mice, and the approach improved learning and memory behaviors across all strains, further supporting the hypothesis that gene therapy via local AAV delivery at the early stages of pathology offers long-term protective effects, preventing disease progression.

We applied AAVT42, a serotype screened in our lab for its high tropism to skeletal muscle. In this study, we first characterized its tropism for neurons in adult WT and AD model mice, as well as aged non-human primates. In contrast, the neuronal transduction efficiency of AAV9 and its variants appears to be model-dependent^72^ and declines with age.^73^ Although systematic AAV9 delivery methods, such as intravenous or intracerebroventricular injections, have been reported to effectively penetrate the blood–brain barrier and achieve widespread distribution throughout the CNS,^74^^,^^75^ and these delivery approaches require higher doses and present safety concerns due to potential toxicity in periphery organs.^76^ Therefore, the intrabrain injection remains the primary AAV delivery method for treating CNS disorders, and AAVT42 showed an advantage for further application in clinical trials targeting neurons.

When comparing the therapeutic effects across three AD mouse models, BDNF overexpression improved performance in the Y-maze test for both APP/PS1 and rTg4510 mice, indicating a restoration of short-term spatial working memory.^77^ Consistently, improved performance was observed in the Morris water maze and Barnes maze tests in 3 × Tg mice, further confirming that the therapeutic effect of BDNF overexpression is independent of Aβ and/or p-tau features in all the tested AD model mice. During the water maze test, the treated APP/PS1 mice performed better, while rTg4510 mice showed a trend toward improved learning ability during training, although this improvement did not reach statistical significance, which might be caused by significant neuronal loss and hippocampal atrophy in rTg4510 mice at early adulthood, with BDNF administration could only partially improve spatial memory. In the conditioning fear test, the treated rTg4510 mice exhibited better context memory, underscoring the critical role of BDNF in contextual fear conditioning.^78^ All groups showed strong freezing responses to auditory stimuli in the cued test as all mice could associate the noise with the electronic shock finally, suggesting that rTg4510 mice did not show obvious deficits in processing emotional and stress responses, which involve multiple brain regions, including amygdala, hypothalamus, and prefrontal cortex.

Additionally, we observed increased locomotor activities in APP/PS1 mice following AAVT42-BDNF administration, as evidenced by increased total travel distance and velocity in the open field test as well as increased entry frequency in the Y-maze. These findings strongly suggest reduced anxiety after BDNF overexpression, aligning with previous studies that BDNF overexpression improves stress resilience, which is positively correlated with enhanced hippocampal neurogenesis.^79^ In rTg4510 mice, however, BDNF administration did not significantly alter locomotor activities, likely due to the pronounced hyperactivity inherent in these bi-transgenic mice,^46^ which involves multiple brain regions beyond the hippocampus.^46^ Taken together, the impact of enhanced locomotor activities on AD rodents, particularly in the context of neurodegenerative disease, requires further evaluation.

BDNF treatment in 3 × Tg mice down-regulated expression of *Bmps,* indicating that enhanced neurogenesis may inhibit downstream BMP signaling or require the inhibition of BMP signaling. However, BMP expression did not change in APP/PS1 mice, and there was no significant reduction after BDNF treatment in rTg4510 mice even though rTg4510 mice had higher levels of *Bmps* compared to wild type littermates. This inconsistency reflects the complexity of BMP signaling in AD mouse models and the progression of the disease. For example, BMP4 overexpression in transgenic mice impairs memory and increases Aβ and tau accumulation,^67^ whereas lower levels of BMP6 in AD patients are associated with cognitive impairment.^80^ BMP7 exhibits neuroprotective effects against Aβ-induced neurotoxicity *in vitro*.^81^ These findings suggest that BMP signaling is intricately involved in the pathology and potential treatment of AD, with its regulation by BDNF potentially differing based on Aβ or p-tau accumulation.

BDNF's neuronal effects are mediated through interactions with its receptors, TrkB and p75, which are disrupted by Aβ or tau pathology.^82^ Our study further proved the primary functions of BDNF in neuroprotection and synaptic plasticity against Aβ and tau-induced neurotoxicity, while they had no direct impact on amyloidosis or tau accumulation. The possible explanation is that BDNF administration specifically in the hippocampus interacts with various signaling ways involved in synaptic plasticity, which are not directly involved in amyloid metabolism^83^ or tau phosphorylation.^47^ Besides, neuroinflammation plays a critical role in amyloid clearance, and our results showed that BDNF did not modulate neuroinflammation effectively. In our ongoing work, we are creating vectors carrying BDNF and the pro-inflammatory inhibitor interleukin-1 receptor antagonist (IL-1RA) to explore the combined therapy effect.

In clinical trials for AD, the delivery of the nerve growth factor via AAV2 to the nucleus basalis of Meynert has demonstrated long-term safety, however, challenges include inefficient delivery to target neurons and potential side effects like nociceptive responses.^30^^,^^84^ Similarly, BDNF gene therapy has been investigated for various neurodegenerative diseases, including Parkinson's, Alzheimer's, and Huntington's diseases.^85^^,^^86^ Currently, BDNF administration for AD in clinical trials is ongoing, with a focus on the regulation of BDNF expression and the delivery: precise regulation of BDNF expression is essential, as both overload and insufficiency can cause adverse effects.^87^^,^^88^ Additionally, effective delivery of BDNF across the blood–brain barrier remains a significant challenge. Our results support the use of AAV-based gene therapy for AD through the local delivery of BDNF to the hippocampus, which has the highest level of BDNF mRNA in the CNS and is also assumed to have a critical role in the early clinical manifestations of AD.^89^^,^^90^ We believe that the novel rAAV vectors, exhibiting high transduction efficiency and safer profiles, along with optimized delivery routines and surgical management, will facilitate the application of AAVs in treating neurological disorders in the future.

## CRediT authorship contribution statement

**Siqi Tang:** Writing – review & editing, Writing – original draft, Validation, Software, Methodology, Investigation, Data curation. **Wenshu Luo:** Writing – review & editing, Software, Methodology, Formal analysis. **Shihao Wu:** Writing – review & editing, Methodology. **Meng Yuan:** Data curation, Writing – review & editing. **Jiashuo Wen:** Data curation, Writing – review & editing. **Guoshen Zhong:** Data curation, Writing – review & editing. **Leshan Shen:** Writing – review & editing, Data curation. **Wei Jiang:** Resources, Methodology. **Cheng Cheng:** Writing – review & editing, Methodology, Investigation, Data curation. **Xia Wu:** Supervision, Funding acquisition. **Xiao Xiao:** Supervision, Resources, Investigation, Funding acquisition, Data curation, Conceptualization.

## Data availability

The datasets used and/or analyzed during the study are available upon reasonable request from the corresponding author.

## Funding

This work was supported by the 10.13039/501100012166National Key Research and Development Program of China (No. 2021YFC2700803) and the 10.13039/501100001809National Natural Science Foundation of China (No.82471501, 82360226).

## Conflict of interests

Xiao Xiao is the inventor of the patent about the AAVT42 serotype used in this paper (reference to patent No. US 2016/0304904 A1). Wenshu Luo and Wei Jiang currently work for Belief Biomed Inc, and XiaoXiao is the company's founder.

## References

[1] Zemek F., Drtinova L., Nepovimova E. (2014). Outcomes of Alzheimer's disease therapy with acetylcholinesterase inhibitors and memantine. Expet Opin Drug Saf.

[2] Mufson E.J., Counts S.E., Perez S.E., Ginsberg S.D. (2008). Cholinergic system during the progression of Alzheimer's disease: therapeutic implications. Expert Rev Neurother.

[3] Thal D.R., Rüb U., Orantes M., Braak H. (2002). Phases of A beta-deposition in the human brain and its relevance for the development of AD. Neurology.

[4] Braak H., Braak E. (1991). Neuropathological stageing of alzheimer-related changes. Acta Neuropathol.

[5] van Dyck C.H., Swanson C.J., Aisen P. (2023). Lecanemab in early Alzheimer's disease. N Engl J Med.

[6] Sims J.R., Zimmer J.A., Evans C.D. (2023). Donanemab in early symptomatic Alzheimer disease: the TRAILBLAZER-ALZ 2 randomized clinical trial. JAMA.

[7] Budd Haeberlein S., Aisen P.S., Barkhof F. (2022). Two randomized phase 3 studies of aducanumab in early Alzheimer's disease. J Prev Alzheimers Dis.

[8] Jucker M., Walker L.C. (2023). Alzheimer's disease: from immunotherapy to immunoprevention. Cell.

[9] Poo M.M. (2001). Neurotrophins as synaptic modulators. Nat Rev Neurosci.

[10] Bramham C.R., Messaoudi E. (2005). BDNF function in adult synaptic plasticity: the synaptic consolidation hypothesis. Prog Neurobiol.

[11] Almeida R.D., Manadas B.J., Melo C.V. (2005). Neuroprotection by BDNF against glutamate-induced apoptotic cell death is mediated by ERK and PI_3_^-^kinase pathways. Cell Death Differ.

[12] Mao X.Y., Zhou H.H., Li X., Liu Z.Q. (2016). Huperzine A alleviates oxidative glutamate toxicity in hippocampal HT22 cells *via* activating BDNF/TrkB-dependent PI3K/Akt/mTOR signaling pathway. Cell Mol Neurobiol.

[13] Laske C., Stransky E., Leyhe T. (2007). BDNF serum and CSF concentrations in Alzheimer's disease, normal pressure *Hydrocephalus* and healthy controls. J Psychiatr Res.

[14] Zhang J., Sokal I., Peskind E.R. (2008). CSF multianalyte profile distinguishes Alzheimer and Parkinson diseases. Am J Clin Pathol.

[15] Diniz B.S., Teixeira A.L. (2011). Brain-derived neurotrophic factor and Alzheimer’s disease: physiopathology and beyond. Neuromolecular Med.

[16] Ventriglia M., Zanardini R., Bonomini C. (2013). Serum brain-derived neurotrophic factor levels in different neurological diseases. Biomed Res Int.

[17] Forlenza O.V., Diniz B.S., Teixeira A.L. (2015). Lower cerebrospinal fluid concentration of brain-derived neurotrophic factor predicts progression from mild cognitive impairment to Alzheimer’s disease. Neuromolecular Med.

[18] Iulita M.F., Bistué Millón M.B., Pentz R. (2017). Differential deregulation of NGF and BDNF neurotrophins in a transgenic rat model of Alzheimer's disease. Neurobiol Dis.

[19] Quesseveur G., David D.J., Gaillard M.C. (2013). BDNF overexpression in mouse hippocampal astrocytes promotes local neurogenesis and elicits anxiolytic-like activities. Transl Psychiatry.

[20] de Pins B., Cifuentes-Díaz C., Farah A.T. (2019). Conditional BDNF delivery from astrocytes rescues memory deficits, spine density, and synaptic properties in the 5xFAD mouse model of Alzheimer disease. J Neurosci.

[21] Caffino L., Mottarlini F., Fumagalli F. (2020). Born to protect: leveraging BDNF against cognitive deficit in Alzheimer's disease. CNS Drugs.

[22] Kuzmin D.A., Shutova M.V., Johnston N.R. (2021). The clinical landscape for AAV gene therapies. Nat Rev Drug Discov.

[23] Deverman B.E., Ravina B.M., Bankiewicz K.S., Paul S.M., Sah D.W.Y. (2018). Gene therapy for neurological disorders: progress and prospects. Nat Rev Drug Discov.

[24] Keiser M.S., Chen Y.H., Davidson B.L. (2018). Techniques for intracranial stereotaxic injections of adeno-associated viral vectors in adult mice. Curr Protoc Mouse Biol.

[25] Kang L., Jin S., Wang J. (2023). AAV vectors applied to the treatment of CNS disorders: clinical status and challenges. J Control Release.

[26] Gonzalez T.J., Mitchell-Dick A., Blondel L.O. (2023). Structure-guided AAV capsid evolution strategies for enhanced CNS gene delivery. Nat Protoc.

[27] Shen W., Liu S., Ou L. (2022). rAAV immunogenicity, toxicity, and durability in 255 clinical trials: a meta-analysis. Front Immunol.

[28] Ling Q., Herstine J.A., Bradbury A., Gray S.J. (2023). AAV-based *in vivo* gene therapy for neurological disorders. Nat Rev Drug Discov.

[29] Tuszynski M.H., Yang J.H., Barba D. (2015). Nerve growth factor gene therapy: activation of neuronal responses in Alzheimer disease. JAMA Neurol.

[30] Rafii M.S., Tuszynski M.H., Thomas R.G. (2018). Adeno-associated viral vector (serotype 2)-nerve growth factor for patients with Alzheimer disease: a randomized clinical trial. JAMA Neurol.

[31] Liu Z., Ma D., Feng G., Ma Y., Hu H. (2007). Recombinant AAV-mediated expression of human BDNF protects neurons against cell apoptosis in Aβ-induced neuronal damage model. J Huazhong Univ Sci Technolog Med Sci.

[32] Nagahara A.H., Merrill D.A., Coppola G. (2009). Neuroprotective effects of brain-derived neurotrophic factor in rodent and primate models of Alzheimer's disease. Nat Med.

[33] Yanagisawa D., Hamezah H.S., Pahrudin Arrozi A., Tooyama I. (2021). Differential accumulation of tau pathology between reciprocal F1 hybrids of rTg4510 mice. Sci Rep.

[34] Yanagisawa D., Hamezah H.S., Durani L.W., Taguchi H., Tooyama I. (2018). Study of tau pathology in male rTg4510 mice fed with a curcumin derivative Shiga-Y5. PLoS One.

[35] Wang Y., Yang C., Hu H. (2022). Directed evolution of adeno-associated virus 5 capsid enables specific liver tropism. Mol Ther Nucleic Acids.

[36] Cai M., Lee J.H., Yang E.J. (2019). Electroacupuncture attenuates cognition impairment *via* anti-neuroinflammation in an Alzheimer's disease animal model. J Neuroinflammation.

[37] Miedel C.J., Patton J.M., Miedel A.N., Miedel E.S., Levenson J.M. (2017). Assessment of spontaneous alternation, novel object recognition and limb clasping in transgenic mouse models of amyloid-β and tau neuropathology. J Vis Exp.

[38] Bromley-Brits K., Deng Y., Song W. (2011). Morris water maze test for learning and memory deficits in Alzheimer's disease model mice. J Vis Exp.

[39] Ferreira T.A., Blackman A.V., Oyrer J. (2014). Neuronal morphometry directly from bitmap images. Nat Methods.

[40] Yang L., Li J., Xiao X. (2011). Directed evolution of adeno-associated virus (AAV) as vector for muscle gene therapy. Methods Mol Biol.

[41] Johnson G.V., Jope R.S. (1992). The role of microtubule-associated protein 2 (MAP-2) in neuronal growth, plasticity, and degeneration. J Neurosci Res.

[42] Ma X., Cheng O., Jiang Q., Yang J., Xiao H., Qiu H. (2021). Activation of ephrinb1/EPHB2/MAP-2/NMDAR mediates hippocampal neurogenesis promoted by transcranial direct current stimulation in cerebral-ischemic mice. NeuroMolecular Med.

[43] Wegmann S., Biernat J., Mandelkow E. (2021). A current view on Tau protein phosphorylation in Alzheimer's disease. Curr Opin Neurobiol.

[44] Helboe L., Egebjerg J., Barkholt P., Volbracht C. (2017). Early depletion of CA1 neurons and late neurodegeneration in a mouse tauopathy model. Brain Res.

[45] Anglada-Huguet M., Rodrigues S., Hochgräfe K., Mandelkow E., Mandelkow E.M. (2021). Inhibition of Tau aggregation with BSc3094 reduces Tau and decreases cognitive deficits in rTg4510 mice. Alzheimers Dement (N Y).

[46] Wang X., Smith K., Pearson M. (2018). Early intervention of tau pathology prevents behavioral changes in the rTg4510 mouse model of tauopathy. PLoS One.

[47] Jiao S.S., Shen L.L., Zhu C. (2016). Brain-derived neurotrophic factor protects against tau-related neurodegeneration of Alzheimer's disease. Transl Psychiatry.

[48] Sahara N., Perez P.D., Lin W.L. (2014). Age-related decline in white matter integrity in a mouse model of tauopathy: an *in vivo* diffusion tensor magnetic resonance imaging study. Neurobiol Aging.

[49] Blair L.J., Frauen H.D., Zhang B. (2015). Tau depletion prevents progressive blood-brain barrier damage in a mouse model of tauopathy. Acta Neuropathol Commun.

[50] Hernandez I., Luna G., Rauch J.N. (2019). A farnesyltransferase inhibitor activates lysosomes and reduces tau pathology in mice with tauopathy. Sci Transl Med.

[51] Quinn J.P., Kandigian S.E., Trombetta B.A., Arnold S.E., Carlyle B.C. (2021). VGF as a biomarker and therapeutic target in neurodegenerative and psychiatric diseases. Brain Commun.

[52] Shi R., Redman P., Ghose D., et al. Shank proteins differentially regulate synaptic transmission. eNeuro. 2017;4(6):163:ENEURO.0163-15.2017. 10.1523/ENEURO.0163-15.2017 PMC5731535 29250591

[53] Goshima Y., Yamashita N., Nakamura F., Sasaki Y. (2016). Regulation of dendritic development by semaphorin 3A through novel intracellular remote signaling. Cell Adhes Migrat.

[54] Tadros W., Xu S., Akin O. (2016). Dscam proteins direct dendritic targeting through adhesion. Neuron.

[55] Song M., Giza J., Proenca C.C. (2015). Slitrk5 mediates BDNF-dependent TrkB receptor trafficking and signaling. Dev Cell.

[56] Sachse C.C., Kim Y.H., Agsten M. (2013). BACE1 and presenilin/γ-secretase regulate proteolytic processing of KCNE1 and 2, auxiliary subunits of voltage-gated potassium channels. FASEB J.

[57] Mullins R., Kapogiannis D. (2022). Alzheimer's disease-related genes identified by linking spatial patterns of pathology and gene expression. Front Neurosci.

[58] Ren J., Zhang B., Wei D., Zhang Z. (2020). Identification of methylated gene biomarkers in patients with Alzheimer's disease based on machine learning. BioMed Res Int.

[59] Quitterer U., AbdAlla S. (2020). Improvements of symptoms of Alzheimer's disease by inhibition of the angiotensin system. Pharmacol Res.

[60] Pietronigro E., Zenaro E., Bianca V.D. (2019). Blockade of α4 integrins reduces leukocyte-endothelial interactions in cerebral vessels and improves memory in a mouse model of Alzheimer's disease. Sci Rep.

[61] Manzari-Tavakoli A., Babajani A., Farjoo M.H., Hajinasrollah M., Bahrami S., Niknejad H. (2022). The cross-talks among bone morphogenetic protein (BMP) signaling and other prominent pathways involved in neural differentiation. Front Mol Neurosci.

[62] Li C., Wu X., Liu S., Zhao Y., Zhu J., Liu K. (2019). Roles of neuropeptide Y in neurodegenerative and neuroimmune diseases. Front Neurosci.

[63] Pedersen W.A., McCullers D., Culmsee C., Haughey N.J., Herman J.P., Mattson M.P. (2001). Corticotropin-releasing hormone protects neurons against insults relevant to the pathogenesis of Alzheimer's disease. Neurobiol Dis.

[64] Adachi N., Suzuki S., Matsuoka H. (2018). Corticotropin-releasing hormone-binding protein is up-regulated by brain-derived neurotrophic factor and is secreted in an activity-dependent manner in rat cerebral cortical neurons. J Neurochem.

[65] Dong B.E., Chen H., Sakata K. (2020). *BDNF* deficiency and enriched environment treatment affect neurotransmitter gene expression differently across ages. J Neurochem.

[66] Zhu M., Tang M., Du Y. (2023). Identification of TAC1 associated with Alzheimer's disease using a robust rank aggregation approach. J Alzheimers Dis.

[67] Zhang X., Li J., Ma L. (2021). BMP4 overexpression induces the upregulation of APP/Tau and memory deficits in Alzheimer's disease. Cell Death Discov.

[68] Crews L., Adame A., Patrick C. (2010). Increased BMP6 levels in the brains of Alzheimer's disease patients and APP transgenic mice are accompanied by impaired neurogenesis. J Neurosci.

[69] Tunc-Ozcan E., Brooker S.M., Bonds J.A. (2021). Hippocampal BMP signaling as a common pathway for antidepressant action. Cell Mol Life Sci.

[70] Chen W.W., Fu W.Y., Su Y.T., Fang W.Q., Fu A.K.Y., Ip N.Y. (2019). Increased Axin expression enhances adult hippocampal neurogenesis and exerts an antidepressant effect. Sci Rep.

[71] Gao Z., Houthuijzen J.M., Ten Dijke P., Brazil D.P. (2023). GREM1 signaling in cancer: tumor promotor and suppressor?. J Cell Commun Signal.

[72] Hordeaux J., Wang Q., Katz N., Buza E.L., Bell P., Wilson J.M. (2018). The neurotropic properties of AAV-PHP.B are limited to C57BL/6J mice. Mol Ther.

[73] Saraiva J., Nobre R.J., Pereira de Almeida L. (2016). Gene therapy for the CNS using AAVs: the impact of systemic delivery by AAV9. J Contr Release.

[74] Chan K.Y., Jang M.J., Yoo B.B. (2017). Engineered AAVs for efficient noninvasive gene delivery to the central and peripheral nervous systems. Nat Neurosci.

[75] Deverman B.E., Pravdo P.L., Simpson B.P. (2016). Cre-dependent selection yields AAV variants for widespread gene transfer to the adult brain. Nat Biotechnol.

[76] Lek A., Wong B., Keeler A. (2023). Death after high-dose rAAV9 gene therapy in a patient with Duchenne's muscular dystrophy. N Engl J Med.

[77] Prieur E.A.K., Jadavji N.M. (2019). Assessing spatial working memory using the spontaneous alternation Y-maze test in aged male mice. Bio Protoc.

[78] Liu I.Y.C., Ernest Lyons W., Mamounas L.A., Thompson R.F. (2004). Brain-derived neurotrophic factor plays a critical role in contextual fear conditioning. J Neurosci.

[79] Leschik J., Gentile A., Cicek C. (2022). Brain-derived neurotrophic factor expression in serotonergic neurons improves stress resilience and promotes adult hippocampal neurogenesis. Prog Neurobiol.

[80] Sun L., Guo C., Song Y., Sheng J., Xiao S. (2022). Alzheimer's Disease Neuroimaging Initiative. Blood BMP6 associated with cognitive performance and Alzheimer's disease diagnosis: a longitudinal study of Elders. J Alzheimers Dis.

[81] Sun L., Guo C., Liu D. (2011). Protective effects of bone morphogenetic protein 7 against amyloid-beta induced neurotoxicity in PC12 cells. Neuroscience.

[82] Eggert S., Kins S., Endres K., Brigadski T. (2021). Brothers in arms: probdnf/bdnf and sAPPα/Aβ-signaling and their common interplay with ADAM10, TrkB, p75NTR, sortilin, and sorLA in the progression of Alzheimer's disease. Biol Chem.

[83] Psotta L., Rockahr C., Gruss M. (2015). Impact of an additional chronic BDNF reduction on learning performance in an Alzheimer mouse model. Front Behav Neurosci.

[84] Mandel R.J. (2010). CERE-110, an adeno-associated virus-based gene delivery vector expressing human nerve growth factor for the treatment of Alzheimer's disease. Curr Opin Mol Therapeut.

[85] Zhao H., Alam A., San C.Y. (2017). Molecular mechanisms of brain-derived neurotrophic factor in neuro-protection: recent developments. Brain Res.

[86] Katsu-Jiménez Y., Loría F., Corona J.C., Díaz-Nido J. (2016). Gene transfer of brain-derived neurotrophic factor (BDNF) prevents neurodegeneration triggered by FXN deficiency. Mol Ther.

[87] Martínez-Levy G.A., Cruz-Fuentes C.S. (2014). Genetic and epigenetic regulation of the brain-derived neurotrophic factor in the central nervous system. Yale J Biol Med.

[88] Eyileten C., Sharif L., Wicik Z. (2021). The relation of the brain-derived neurotrophic factor with microRNAs in neurodegenerative diseases and ischemic stroke. Mol Neurobiol.

[89] Mu Y., Gage F.H. (2011). Adult hippocampal neurogenesis and its role in Alzheimer's disease. Mol Neurodegener.

[90] Liu D., Zhu M., Zhang Y., Diao Y. (2021). Crossing the blood-brain barrier with AAV vectors. Metab Brain Dis.

