# Supplementary material for: Hippocampus-targeted BDNF gene therapy to rescue cognitive impairments of Alzheimer's disease in multiple mouse models
Source: Genes Dis. 2025 Apr 22;13(2):101649. doi: 10.1016/j.gendis.2025.101649 (PMC12754220; doi:10.1016/j.gendis.2025.101649)
Supplement: Multimedia component 1 [file mmc1.docx]

**Supplementary Figures**

**
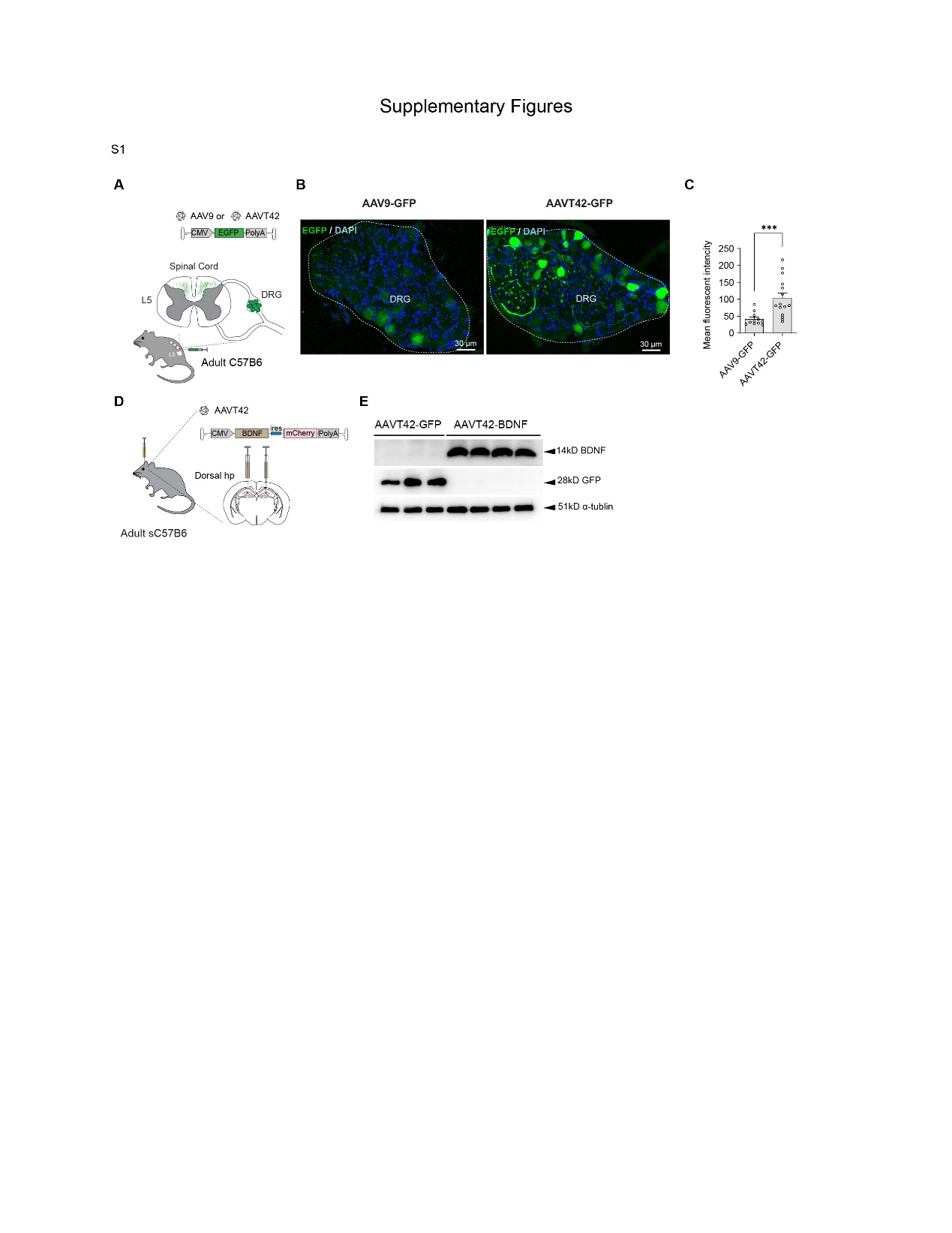
**

**Supplementary Figure 1 AAVT42 transfection efficiency in periphery nervous system and long-term BDNF expression after AAVT42-BDNF injection in the adult mice hippocampus**

1. Schematic diagram of intrathecal AAV injection. AAV-vectors were injected into lower tip intrathecal site. Total 20 μL of 4×10^12 GC/mL of viruses were injected into L5-vertebrae and resulted in vector transduction of DRGs along the lumbar enlargement.
2. Representative confocal microscope images of EGFP expression in DRGs after 4 weeks injection.

C. Quantification analysis of EGFP fluorescent intensity. Mean intensity of EGFP signaling in each transduced DRGs’ soma: AAV9-GFP = 41.22 ± 5.78, n = 11 cells; AAVT42-GFP = 103.8 ±14.79, n = 15 cells. ***p=0.0005.

D and E. Western blot analysis for transgenic BDNF expression after AAVT42-BDNF injection in hippocampus of C57 mice. Tissues were collected 4 months after injection.


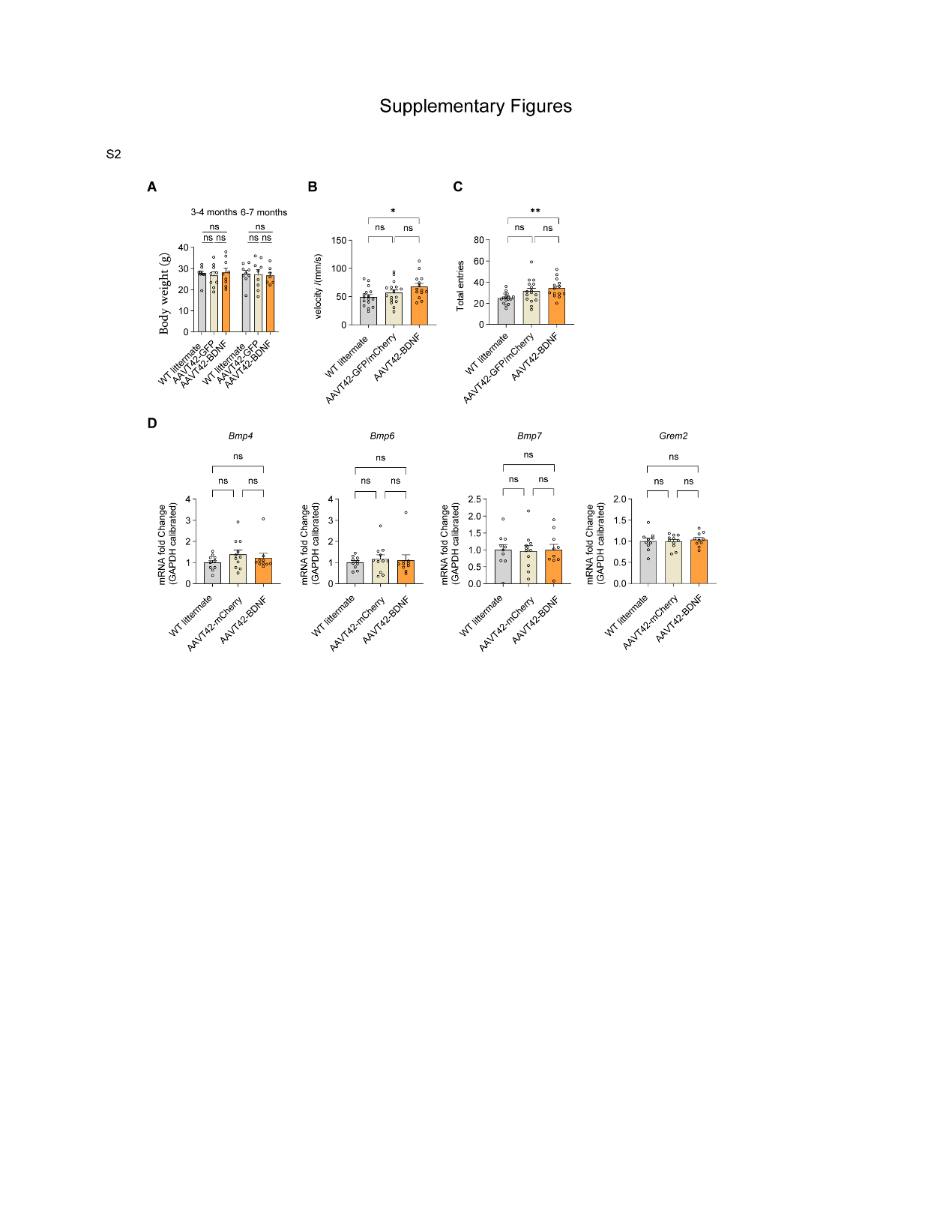


**Supplementary Figure 2 Body weight and qPCR results in APP/PS1 mice**

A. Body weight of a batch of APP/PS1 mice. WT littermate, n = 9; AAVT42-GFP, n = 9; AAVT42-BDNF, n = 7. Before injection at 3-4 months, WT littermate: 27.57 ± 1.14 g; AAVT42-GFP: 26.69 ± 1.80 g; AAVT42-BDNF: 27.83 ± 1.93g.  *P* > 0.99 for WT littermate vs AAVT42-GFP, *P* = 0.61 for AAVT42-GFP vs. AAVT42-BDNF, *P* = 0.95 for WT littermate vs. AAVT42-BDNF. After injection at the 6-7 months, WT littermate: 27.54 ± 1.55 g; AAVT42-GFP: 27.24 ± 2.27 g; AAVT42-BDNF: 26.69 ± 1.57. *P* > 0.99 for WT littermate vs AAVT42-GFP, *P* = 0.78 for AAVT42-GFP vs. AAVT42-BDNF, *P* = 0.55 for WT littermate vs. AAVT42-BDNF.

B. Velocity during an open field test of APP/PS1 mice. WT littermate: 49.30 ± 4.24mm/s, AAVT42-GFP/mCherry: 56.70 ± 4.95 mm/s; AAVT42-BDNF:67.58 ± 5.62 mm/s. *P* = 0.28 for WT littermate vs. AAVT42-GFP/mCherry, *P* = 0.13 for AAVT42-GFP/mCherry vs. AAVT42-BDNF, **P* = 0.013 for WT littermate vs. AAVT42-BDNF.

C. Total entry times during the Y-maze test of APP/PS1 mice. WT littermate: 24.75 ± 1.32; AAVT42-GFP/mCherry: 31.44 ± 2.77; AAVT42-BDNF: 34.77 ± 2.46. *P* = 0.036 for WT littermate vs. AAVT42-GFP/mCherry, *P* = 0.31 for AAVT42-GFP/mCherry vs. AAVT42-BDNF; ***P* = 3.7×10^-3^ for WT littermate vs. AAVT42-BDNF.

D Genes involved in the BMP signaling pathway were further detected by real-time qPCR. n≥10 for each group. Each point represents the result from half of the hippocampus. Low *Grem1* expression level was not detected by qPCR.


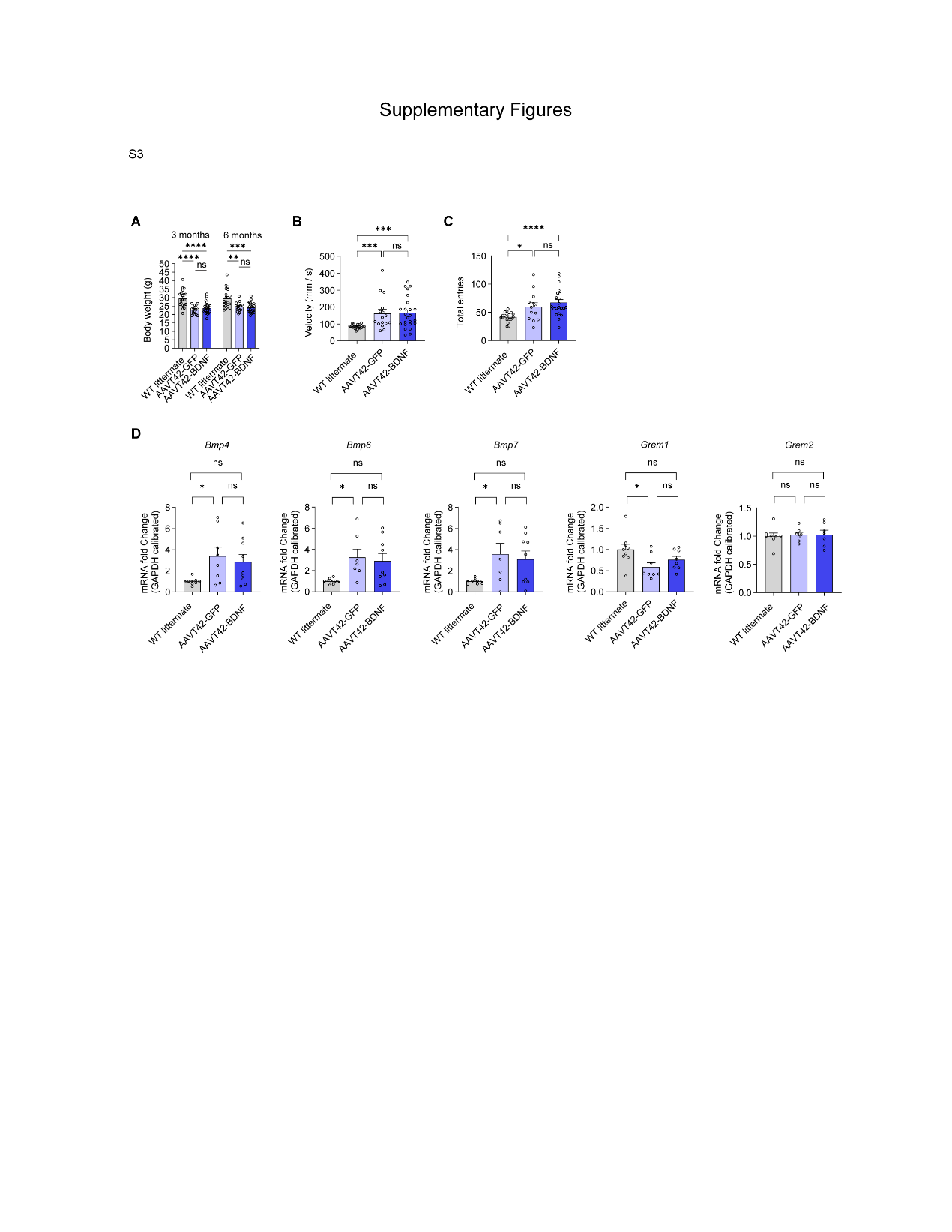


**Supplementary Figure 3 Body weight and qPCR results in rTg4510 mice**

A. Body weight of two batches of rTg4510 mice. WT littermate, n = 20; AAVT42-GFP, n = 16; AAVT42-BDNF, n = 24. Before injection at 3 months, WT littermate: 29.44 ± 1.12 g; AAVT42-GFP: 22.81 ± 0.62 g; AAVT42-BDNF = 23.78 ± 0.68g. *****P* =2.7×10^-5^ for WT littermate vs. AAVT42-GFP, *P* = 0.33 for AAVT42-GFP vs. AAVT42-BDNF, *****P* =5.4×10^-5^ for WT littermate vs. AAVT42-BDNF. After injection at 6 months, WT littermate: 29.47 ± 1.24 g; AAVT42-GFP: 24.40 ± 0.66 g; AAVT42-BDNF: 24.00 ± 0.60 g. ***P* = 1.9×10^-3^ for WT littermate vs. AAVT42-GFP, *P* = 0.66 for AAVT42-GFP vs. AAVT42-BDNF, ****P* = 1.4×10^-4^ for WT littermate vs. AAVT42-BDNF.

B. Velocity during the open field test of rTg4510 mice. WT littermate: 84.84 ± 2.70 mm/s; AAVT42-GFP = 161.7 ± 24.2 mm/s; AAVT42-BDNF: 164.7 ± 18.9 mm/s. ****P* = 8.0×10^-3^ for WT littermate vs. AAVT42-GFP, *P* = 0.93 for AAVT42-GFP vs. AAVT42-BDNF, ****P* = 1.0×10^-4^ for WT littermate vs. AAVT42-BDNF.

C. Total entry times during the Y-maze test of rTg4510 mice. WT littermate: 41.75 ± 1.80; AAVT42-GFP = 59.85 ± 7.23; AAVT42-BDNF = 67.33 ± 5.37. **P* = 0.018 for WT littermate vs. AAVT42-GFP, *P* = 0.28 for AAVT42-GFP vs. AAVT42-BDNF, *****P* < 1.0×10^-4^ for WT littermate vs. AAVT42-BDNF.

D. Genes involved in the BMP signaling pathway were further detected by real-time qPCR. n≥7 for each group. Each point represents the result from half of the hippocampus. WT littermate vs. AAVT42-GFP, **P* = 0.041 for *Bmp4*, **P* = 0.039 for *Bmp6*, **P* = 0.045 for *Bmp7*, **P* = 0.029 for *Grem1*.


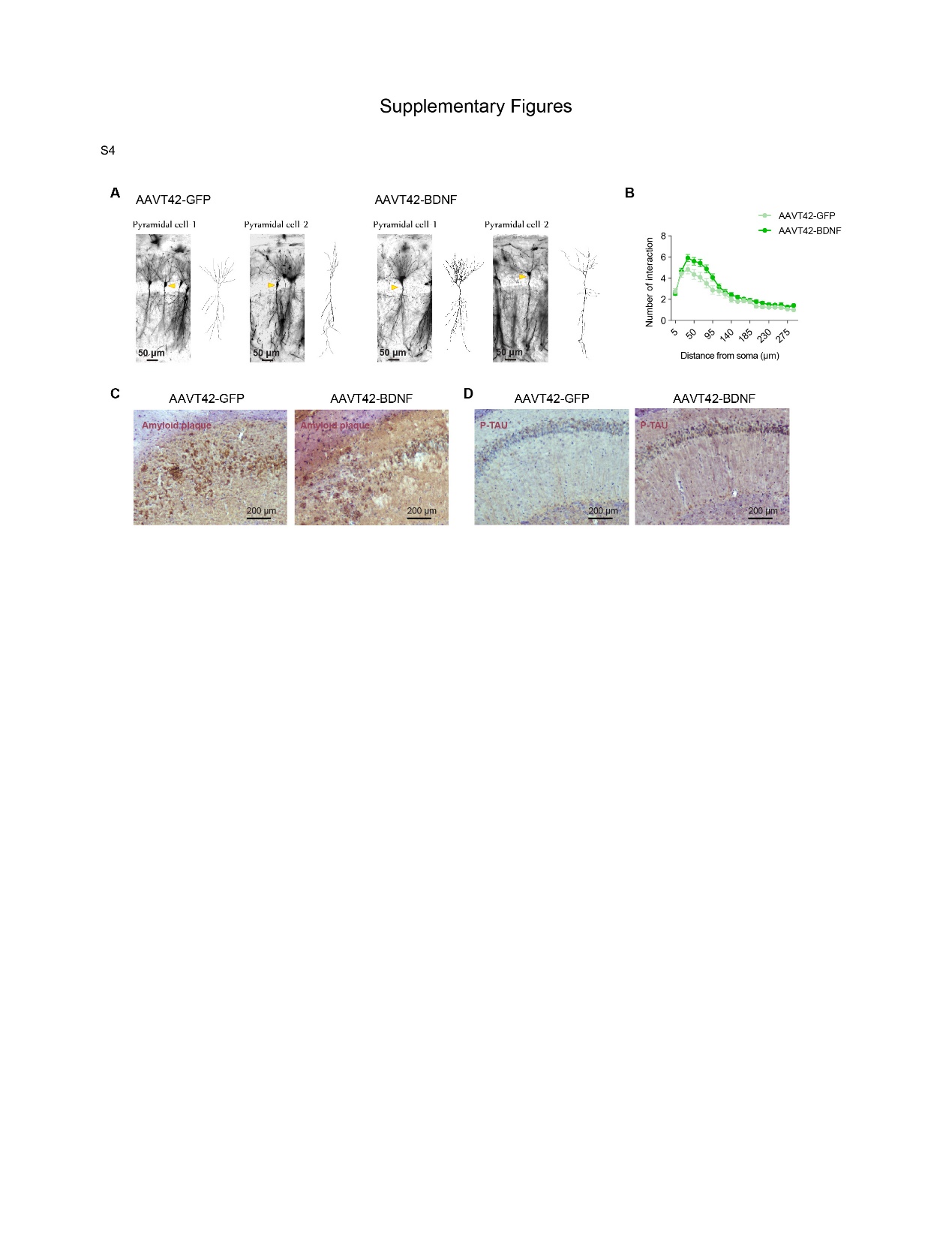


**Supplementary Figure 4 Dendritic analysis of traced pyramidal cells and accumulated biomarkers in 3◊Tg mice**

A. Golgi stained pyramidal cells in the CA1 region. The yellow arrows indicated cells for tracing. The corresponding tracing morphology of each pyramidal cell was demonstrated on the right panel.

B. Sholl analysis of traced pyramidal cells. Multiple comparisons of the number of interactions within 5 to 300 µm regions of neurons（n=2 mice of AAVT42-GFP， a total of 38 cells were analyzed; n=3 mice of AAVT42-BDNF, a total of 50 cells were analyzed Significant results were shown between 35-95 µm from soma.

C and D. Representative images of Aβ (4G8) and phosphorylated Tau (AT8) DAB staining in the hippocampus.
